# Genetic strategies for therapy of Duchenne muscular dystrophy

**DOI:** 10.1016/j.omtn.2025.102759

**Published:** 2025-10-31

**Authors:** Agnieszka Łoboda, Jeffrey S. Chamberlain, Józef Dulak

**Affiliations:** 1 Department of Medical Biotechnology, Faculty of Biochemistry, Biophysics and Biotechnology, Jagiellonian University in Kraków, Kraków, Poland; 2 Department of Neurology, University of Washington School of Medicine & Senator Paul D. Wellstone Muscular Dystrophy Specialized Research Center; Department of Biochemistry; Center for Translational Muscle Research, University of Washington, Seattle, WA, USA

**Keywords:** MT: Delivery Strategies, AAV vectors, antisense oligonucleotide, CRISPR/Cas9, exon skipping, gene editing, gene therapy, split intein, micro-dystrophin, mini-dystrophin, utrophin

## Abstract

Duchenne muscular dystrophy (DMD) is a severe, X-linked genetic disorder caused by mutations in the *DMD* gene, which encodes dystrophin, an essential structural muscle protein. Currently, there are no cures for DMD, and available therapies primarily focus on alleviating symptoms rather than correcting the underlying genetic defect. However, restoration of a shortened version of dystrophin offers the potential for partially addressing the underlying cause of the disease. This review focuses on the promises and challenges of various genetic strategies, such as exon skipping, gene replacement, and gene editing (e.g., by CRISPR-Cas9) aimed at restoring or replacing the dystrophin expression or upregulating utrophin, a paralog of dystrophin that is primarily expressed during fetal life. Finally, novel approaches for modulatory therapies are considered. While they cannot address the cause of DMD, they offer the potential to attenuate the wide-ranging consequences of dystrophin deficiency. Although some of these interventions have demonstrated encouraging preclinical results and early-stage clinical success, challenges remain in optimizing delivery methods, addressing immune responses, and ensuring long-term therapeutic efficacy. Achieving the latter will be crucial for demonstrating the effectiveness of already registered exon-skipping strategies and gene therapy with microdystrophin, which is of utmost importance for the validity of the field.

## Graphical abstract

**Figure:**
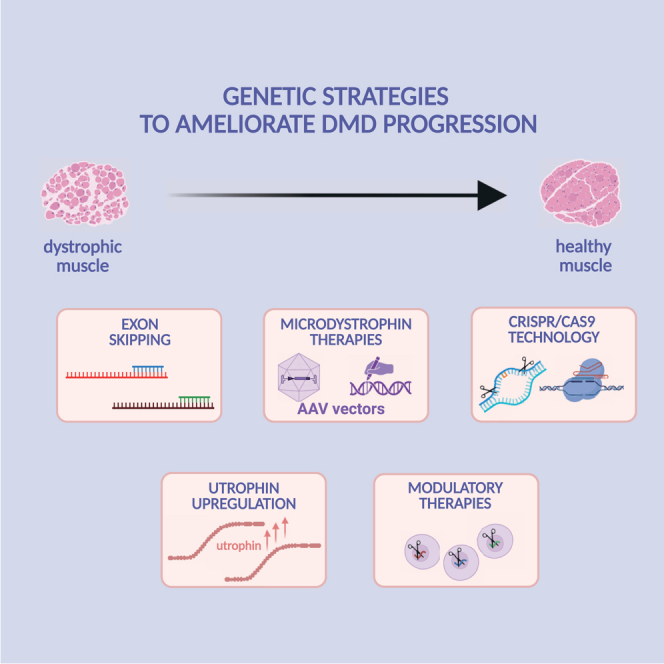

## Teaser

Łoboda, Chamberlain, and Dulak review genetic strategies for Duchenne muscular dystrophy, focusing on exon skipping, gene replacement, and CRISPR-Cas9 editing to restore dystrophin or upregulate utrophin. They also explore modulatory therapies and emphasize key challenges, including delivery methods, immune responses, and sustaining long-term therapeutic efficacy of these approaches.

## Introduction

**Figure 1:**
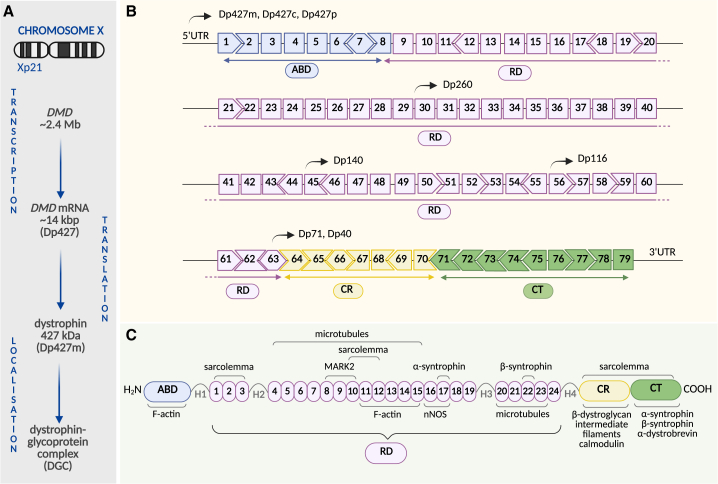
DMD—from gene to protein (A) General overview of the *DMD* processing. (B) The *DMD* gene structure. (C) A simplified structure of the components of dystrophin-glycoprotein complex (DGC) associated with specific domains of dystrophin protein. ABD, actin-binding domain; RD, rod domain; H, hinges; CR, cysteine-rich domain; CT, C-terminal domain.

Duchenne muscular dystrophy (DMD) results from numerous mutations in the *DMD* gene, the largest known gene, spanning about 2.4 million base pairs of genomic DNA. These mutations cause the loss of dystrophin, a structural and scaffolding muscle protein (Figure 1A).^1^ The *DMD* gene is located on the X chromosome (Xp21); therefore, the disease primarily affects boys who inherit a mutated X chromosome from a carrier mother in two-thirds of cases. The remaining cases are due to *de novo* mutations in the single X chromosome that boys possess.^2^ DMD (DMD, MIM #310200) is diagnosed in approximately 1 in 5,000 live male births, making it one of the most frequent forms of muscular dystrophy. Its prevalence is less than 10 cases per 100,000 males.^3^ In contrast, the occurrence of female cases of DMD is exceptionally uncommon (<1 per million); however, some carriers develop generally mild skeletal muscle symptoms such as pain and weakness,^3^ while others may experience issues related to cardiomyopathy (see below). The first cases of this muscle disorder were documented in the early 19th century by Conte, Bell, Partridge, Meryon, Gowers, and Duchenne. Its current name honors Duchenne’s achievements in comprehensively describing the hallmarks of the disease, replacing earlier terms like hypertrophic paraplegia of infancy, pseudo-hypertrophic muscular paralysis, or myo-sclerotic paralysis (reviewed by Tyler^4^). However, the molecular basis of DMD was discovered much later, in the 1980s, when the *DMD* gene was identified and the absence of dystrophin confirmed in muscle biopsies of patients (for references, see review by Hoffman^5^). Furthermore, in 1988, dystrophin was localized on the sarcolemma,^6^ providing further insights into its structural and mechanical role in disease pathology. Since those early discoveries, numerous efforts have been made to identify molecular pathways, discern disease hallmarks, and develop therapies to improve muscle function in DMD patients.

The *DMD* gene (OMIM 300377), discovered in 1986,^7^ consists of 79 primary exons and 78 introns that are expressed in muscle tissues to generate an mRNA of 14,000 nucleotides, although six additional exons are used in non-muscle tissues to generate alternate mRNA isoforms^8^^,^^9^^,^^10^ (Figure 1A). Due to its considerable size, a wide range of mutations can occur in the *DMD* gene; however, the most common types are deletions and duplications. Large-scale analysis revealed that single- or multi-exon intragenic deletions and duplications were detected in approximately 68%–70% and 11%–14% of the cases, respectively. Smaller alterations, accounting for about 20% of mutations, were also identified, including deletions and insertions (of less than one exon), splicing site mutations, point mutations (missense and nonsense), and the rarest intronic ones. Most deletion mutations are predominantly positioned within the so-called hotspots of the *DMD* gene^11^ and cluster around exons 2–20 and 45–55.^1^^,^^3^^,^^12^ Genetic diagnosis of DMD is usually performed using multiplex ligation-dependent probe amplification (MLPA) assays or comparative genome hybridization arrays. These methods enable the identification of deletions and duplications,^13^^,^^14^ while other types of mutations can be detected with next-generation sequencing (NGS).^15^ The *DMD* gene encodes the major, muscle-specific protein, Dp427m, with a molecular weight of 427 kDa and 3,685 amino acids.^8^^,^^9^^,^^16^^,^^17^ This full-length muscle isoform is not the only product synthesized, as the *DMD* locus contains at least seven independent tissue-specific promoters. Three of these promoters, located at the 5′ end of the first exon, direct the transcription of full-length dystrophin isoforms: Dp427m (muscle), Dp427c (cortical, sometimes referred to as Dp427b due to its brain expression), and Dp427p (expressed in cerebellar Purkinje cells). The other, shorter isoforms, transcribed from downstream, internal promoters, are expressed in various, mostly non-muscle, cell types. These latter four promoters, located in introns 29, 44, 55, and 62, produce shorter dystrophin isoforms, Dp260 (retina), Dp140 (central nervous system, kidneys, and embryonic brain), Dp116 (peripheral nerve and Schwann cells, as well as recently reported cardiac tissue^18^), and Dp71 (non-muscle tissue), respectively. Moreover, the Dp40 isoform arises from the same promoter as Dp71, due to the activation of an alternative polyadenylation site in intron 70.^19^^,^^20^^,^^21^ Furthermore, more dystrophin isoforms, such as Dp140ab, Dp140b, Dp140bc, Dp140c, Dp71a, Dp71b, and Dp71ab, are known to exist due to alternative mRNA splicing,^22^^,^^23^^,^^24^ as depicted in Figure 1B. The muscle isoform of dystrophin is composed of four major functional domains: an N-terminal actin-binding domain (ABD); a central coiled-coil segment (rod domain, RD) consisting of 24 spectrin-like repeats (SR) and four hinges (H); a cysteine-rich (CR) domain; and a C-terminal (CT) domain^25^ (Figure 1C).

The specific functions of these domains are closely related to the various proteins associated with dystrophin. These proteins are structural and signaling components of the dystrophin-associated protein complex (DAPC), also known as the dystrophin-glycoprotein complex (DGC), discovered in 1990 by Ervasti et al.^26^ Dystrophin interacts directly with β-dystroglycan (β-DG) through the CR domain, with α- and β-syntrophin via the CT and RD domains, with α-dystrobrevin and neuronal nitric oxide synthase (nNOS) through the CT and RD domains, respectively. In addition, ABD and RD connect dystrophin with cytoskeletal filamentous γ-actin (F-actin). Binding with microtubules occurs within RD, while the CR domain is associated with intermediate filaments and supports the stability of the DGC. Dystrophin has also been suggested to interact directly with microtubule affinity-regulating kinase 2 (MARK2) through the RD and calmodulin in a calcium-dependent manner through the CR domain^3^^,^^27^^,^^28^ (Figure 1C).

## The type of mutation matters—Becker muscular dystrophy

Mutations in the *DMD* gene, found in DMD individuals, are usually frameshift mutations that give rise to premature stop codons in the expressed mRNA. These mutations abolish normal protein translation or result in the formation of non-functional and/or unstable dystrophin. Interestingly, in-frame deletions of *DMD* exon(s) allow the production of a smaller protein with fewer spectrin-like repeats but retaining domains necessary for F-actin and linkage to the extracellular matrix. Importantly, shorter versions of the protein often retain some functionality and are found in patients with Becker muscular dystrophy (BMD, OMIM #300376), a rare type of dystrophy with later onset, milder phenotype, and slower progression. Accordingly, DMD and BMD are generally differentiated by out-of-frame and in-frame mutations, respectively.^29^ However, it is important to note that around 10% of the mutations do not follow the expected predictive pattern. Accordingly, cases of BMD patients with frameshift mutations have been reported, many of which may arise from low-level, natural exon skipping. Similarly, DMD individuals with an mRNA open reading frame have been described. These can arise by removal of in-frame exon(s) that encode critical structural domains, such as the β-dystroglycan binding domain (i.e., CR domain), or in the case of in-frame deletions or missense mutations, which significantly alter dystrophin stability, folding, or association with other proteins such as γ-actin.^19^^,^^30^^,^^31^^,^^32^ Although motor skills may not be significantly impaired in BMD patients, and rare individuals remain ambulant even into their 60s, others are more severely affected and may lose mobility in their teens. Interestingly, during BMD progression, cardiac dysfunction often appears as the first symptom, again indicating the crucial role of dystrophin in proper heart physiology. Respiratory failure and central nervous system abnormalities are rather rarely reported among BMD individuals in contrast to patients with DMD (Table S1).

## Molecular consequences of dystrophin loss

The absence of dystrophin typically results in the loss or low expression of the entire DGC complex, leading to severe impairment of muscle function, dysregulation of muscle fiber mechanical stability during contraction, and disruption of several signaling pathways. These cause segmental myofiber necrosis and membrane leakage while activating the regenerative machinery.^33^^,^^34^ Consequently, repeated cycles of damage and regeneration result in chronic inflammation with unbalanced polarization of so-called M1 and M2 macrophages, increased oxidative stress, and compromised myogenesis. Over time, fibrous and fatty tissues accumulate and replace damaged myofibers, severely impacting muscle function.^35^ Dystrophin deficiency and subsequent dysregulation of the DGC cause significant muscle fiber weakness and increased susceptibility to contraction-induced damage. This ultimately results in the formation of sarcolemmal lesions and membrane leakage,^36^ with the outflow of creatine kinase (CK) and lactate dehydrogenase (LDH) from the muscles,^37^ well-known serum markers of DMD,^38^ and the increase in intracellular Ca^2+^ concentration.^39^^,^^40^ Calcium influx has been demonstrated in both human and animal models.^41^^,^^42^ Furthermore, the loss of dystrophin leads to abnormal activity of various channels, including calcium-permeable channels, due to disturbed interactions between caveolin-3 and β-DG, exacerbating the influx of calcium.^43^ Repeated muscle injuries lead to mitochondrial dysfunction and a significant increase in reactive oxygen species (ROS) generation through dysregulation of the nuclear factor erythroid 2-related factor 2 (NRF2) activity and the expression of its targets, like NADPH oxidase 2 (NOX2) or heme oxygenase-1 (HO-1).^44^

An imbalance in ROS-antioxidant homeostasis activates inflammation via the pro-inflammatory nuclear factor κB (NF-κB) pathway, leading to the activation of neutrophils and macrophages, the major players responsible for the sustained inflammatory phase of the disease. In DMD, continuous cycles of regeneration and degeneration result in the presence of both M1 and M2 macrophages in the muscle at the same time. They simultaneously express pro-inflammatory, pro-fibrotic, and pro-regenerative markers, for example, tumor necrosis factor alpha (TNF-α) and transforming growth factor β (TGF-β).^45^ TGF-β levels are significantly elevated in the muscles of *mdx* mice (which have a point mutation in exon 23 and are the most commonly used mouse model of DMD) and in DMD patients, stimulating the production of ECM proteins such as fibronectin (FN1) and collagens.^46^^,^^47^ In addition to macrophages and neutrophils, cytotoxic CD8^+^, helper CD4^+^, and Treg lymphocytes can contribute to the progression of DMD by inducing necrosis and apoptosis, thereby affecting the biology of muscle satellite cells (mSCs), a population of stem cells in muscles. The imbalance between myogenic differentiation and self-renewal of mSCs, resulting from the repeated cycles of damage and regeneration, was suggested to be associated with the suppression of proliferation, induction of cellular senescence, and telomere shortening.^48^^,^^49^ However, many research groups have reported increased mSCs in dystrophic animals and patients with DMD.^50^^,^^51^ We also found that in *mdx* mice, the defects in regenerative potential are likely not directly related to a reduction in the number of these cells.^52^^,^^53^

As mentioned above, the major cause of death in DMD patients is cardiac abnormalities. In cardiomyocytes, dystrophin absence results in increased structural vulnerability, membrane instability, disruption in Ca^2+^ homeostasis, augmented ROS production, and mitochondrial dysfunction.^54^ Importantly, dysfunction of non-cardiomyocyte cells present in the heart, such as vascular smooth muscle cells, endothelial cells, and fibroblasts, some of which may also express dystrophin,^55^^,^^56^^,^^57^^,^^58^ could contribute to cardiac complications.

## Genetic restoration strategies for dystrophin expression

There is currently no cure for DMD. The majority of DMD patients are given glucocorticoids (prednisone, prednisolone, or deflazacort), which provide beneficial effects on cardiac and skeletal abnormalities and slow progression of the disorder but are associated with a wide range of adverse side effects during long-term use.^59^ A steroidal analog, vamorolone (VBP15), already approved by the Food and Drug Administration (FDA) and European Medicines Agency (EMA), offers comparable anti-inflammatory benefits by slowing muscle degeneration while demonstrating a better safety profile, particularly regarding bone metabolism, bone mineral density, and growth in children.^60^^,^^61^ Moreover, inhibition of histone deacetylase (HDAC) activity, achieved for example by treatment with the pan-HDAC inhibitor givinostat, has emerged as a therapeutic approach in DMD. By modulating gene expression programs, this epigenetic modulator has been shown to reduce fibrosis and inflammation while promoting muscle regeneration and preservation of muscle architecture.^62^ Following the positive outcome of the phase 3 EPIDYS clinical trial (ClinicalTrials.gov, NCT02851797), in which the primary endpoint assessed the difference between givinostat and placebo in the change from baseline to 72 weeks in the four-stair climb test,^63^ the drug subsequently received FDA approval. Although the past decade has brought substantial progress in the field of DMD therapeutics, including the use of cardioprotective agents such as angiotensin-converting enzyme (ACE) inhibitors, beta-blockers, and angiotensin receptor blockers (ARBs) (reviewed in^64^), these treatments primarily serve to improve quality of life and extend life expectancy, but they do not halt the underlying disease course. However, only genetic therapies focus on the main cause of DMD, namely the lack of functional dystrophin. Within this group, readthrough therapy, an antisense oligonucleotide (ASO)-mediated exon skipping approach, vector-mediated gene therapy, and strategies to restore the expression of the shorter form of dystrophin, along with CRISPR/Cas9-mediated gene editing, are under investigation.^65^^,^^66^

Restoration of dystrophin expression is the primary goal of DMD gene therapy. However, this approach faces several challenges. First of all, DMD is the largest known gene, encoding a muscle mRNA, including the untranslated regions, of 13.9 kb (11.2 kb protein-coding sequence).^8^^,^^67^ This prevents loading into adeno-associated viral (AAV) vectors, which have a packaging capacity of only ∼4.7 kb. AAV vectors remain the best option for gene therapy, as they are the only known approach that enables efficient systemic gene delivery to striated muscles.^68^ As mentioned above, dystrophin contains four major functional domains (ABD, RD, CR, and CT). However, it has been found that some internal parts of the dystrophin sequence can be removed with only a modest loss of protein function, and consequently, a series of rod-truncated and CT-domain-lacking dystrophin genes were proposed as alternatives to the full-length protein for use in gene therapy.^69^^,^^70^^,^^71^ This discovery has been possible thanks to elucidating the nature of *DMD* gene mutations in BMD, demonstrating that an artificially truncated version of dystrophin, lacking much of the central rod domain, can be packed even in AAV vectors, creating the chance for *in vivo* gene therapy strategies.^68^^,^^72^^,^^73^ Therefore, the functional but internally deleted “micro”-dystrophin (μDys)^70^^,^^74^ constructs have been developed to facilitate gene transfer (see details below).

Recently, the FDA conditionally approved delandistrogene moxeparvovec (Elevidys), the AAV vector harboring the cDNA for truncated dystrophin, as the therapy for DMD boys between 4 and 5 years of age.^75^^,^^76^^,^^77^ Nevertheless, its efficacy as currently applied is unclear, as the registration was based solely on a surrogate marker, i.e., the expression of microdystrophin. No significant functional improvements have been reported, although the observation period may be too short. Furthermore, the relatively low levels of dystrophin expression achieved to date may be too low for significant long-term efficacy. Additionally, very high costs raise concerns about the sufficient justification for the registration of not-yet-efficient DMD genetic drugs.^75^^,^^78^

Moreover, older, non-ambulant DMD and BMD patients, along with women, the carriers of DMD mutations, are generally not eligible for these therapies. While Sarepta had begun enrolling non-ambulatory patients in their trials, two deaths from acute liver failure ended those studies.^79^ Therefore, there is an utmost need for alternative approaches to ameliorate striated muscle dysfunction. To this end, investigating the molecular mechanisms of dystrophin-dependent cardiomyopathies is critically important, as it can offer insights for better and earlier diagnosis of the disease and uncover potential targets for additional modulatory therapies that could improve patient outcomes and prognosis.

### Exon skipping approach

Clinical observations of BMD patients carrying in-frame exonic deletions and expressing a truncated but partially functional dystrophin^80^ became the basis for the use of ASOs to treat DMD. This approach is also supported by observations in some mutated fibers, where out-of-frame mutations caused by exon deletions are occasionally bypassed naturally through skipping of adjacent exon(s). This process restores the reading frame in the dystrophin mRNA and allows the production of a truncated, partially functional protein. Thus, the ASOs, targeting the splicing of DMD precursor mRNAs, have been tested in animal studies, proving the feasibility of such an approach.

**Figure 2:**
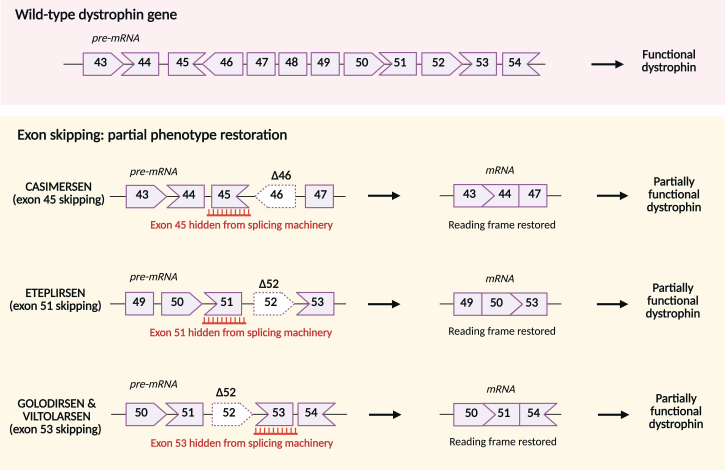
Exon skipping in DMD The mechanism of antisense oligonucleotide (ASOs)-mediated exon skipping approach. Casimersen skips exon 45, Eteplirsen causes exon 51-skipping, while golodirsen and viltolarsen promote skipping of exon 53. The exemplary applications of ASOs in patients with deletion mutation exon 46 (Δ46) and deletion mutation of exon 52 (Δ52) resulting in the formation of truncated but functional dystrophin are shown.

Among various chemical structures, most ASOs for DMD treatment belong to the phosphorodiamidate morpholino oligomers (PMOs).^81^ ASOs bind to the exon/intron boundary or can target intra-exonic regions, leading to exon skipping and restoring the reading frame. Consequently, dystrophin with domains crucial for its activity is produced.^82^ Since 2016, several genetic strategies relying on exon-skipping have been conditionally approved by the FDA. Currently, four ASOs are available for patients with specific mutations (Figure 2). However, the exon-skipping experimental therapies, which rely on ASOs directed toward exons 45, 51, or 53, can only address specific mutations around these exons and not others that cause DMD (reviewed by Łoboda and Dulak^83^). Eteplirsen (or Exondys 51, Sarepta Therapeutics) was the first drug that received accelerated approval for treating DMD patients with a confirmed mutation of the dystrophin gene amenable to exon 51 skipping (Table S2). It leads to restoration of the dystrophin expression lost by the deletion of exon 49/50 but could also work in patients with deletions ending at exon 50 or starting at exon 52 (e.g., 45–50, 47–50, 48–50, 49–50, 50, 52, 52–63).^84^ Accordingly, this drug could be used in about 13%–14% of the population with DMD.^85^^,^^86^

The other drugs, released by Sarepta Therapeutics, include golodirsen (Vyondys 53, SRP-4053) and casimersen (Amondys 45, SRP-4045), enabling *DMD* exon 53 skipping and exon 45 skipping, respectively. They are estimated to be applicable in around 8% of DMD patients, i.e., those with a confirmed exon 53 or 45 amenable mutation. Exon 53 skipping could also be obtained after administering viltolarsen (Viltepso, NS Pharma), which was approved for use in Japan on 25 March 2020.^87^ The drug received an allowance from the FDA later in the same year.

It has to be stressed that all ASOs used currently in the clinics were approved under the accelerated approval pathway. Although meaningful clinical outcomes, such as improvement in the 6-min walk test (6MWT) as a measure of motor function, were not achieved in the clinical trials, these drugs were considered based on surrogate markers, such as increased dystrophin levels/expression in skeletal muscles. The major question is how much dystrophin is necessary and sufficient for normal muscle function; however, the measured amount of dystrophin may vary depending on the evaluation method used (e.g., western blot or immunofluorescent staining).^85^^,^^88^^,^^89^ It is also worth considering if the increase of dystrophin from 0.095% of normal at baseline to 1.019% of normal after 48 weeks of treatment with the golodirsen (assessed by western blot) or from 0.93% at baseline to 1.74% at week 48 among patients treated with 30 mg/kg casimersen once weekly has a real therapeutic impact (for more information and references see Table S2).

In addition to the controversies about the assessment of an increase in dystrophin in muscle biopsy specimens or other methods of evaluating the effectiveness of DMD-targeting ASOs, it should also be underlined that achieving heart-protective effects might be challenging with this approach. As the drugs are administered intravenously, the endosomal entrapment observed in both muscle and cardiac tissues may limit the usefulness of this technology.^90^ The chemical nature of DMD-targeted ASOs (PMO-derived compounds) affects their rapid clearance by the kidneys, as they are uncharged and do not bind serum proteins.^91^ Nevertheless, the effectiveness of ASOs in the heart is much more limited than in the skeletal muscle, with considerable discrepancies observed between muscle and cardiac tissue uptake. Alter et al.^92^ demonstrated that even after multiple injections (seven weekly intravenous injections of ASOs to achieve the maximum cumulative antisense effect), the restoration of dystrophin expression was observed in different muscles but not in cardiac tissue.

This may create a paradox: increased physical activity among DMD patients may lead to further deterioration of heart function if only the muscle functionality is improved by exon skipping.^90^^,^^93^ Therefore, it remains an open question how to enhance the cardiac-related benefits of ASOs. While direct myocardial delivery could theoretically achieve this, it is unlikely to be feasible in humans. To this end, chemical modifications of ASOs, like conjugation with nanoparticles, peptides, and polymers, may help overcome this challenge (reviewed by Nguyen and Yokota^94^). In dystrophic mice, the conjugation of PMOs to peptides (PPMOs) increased cell permeability, resulting in notably higher dystrophin expression in the hearts.^95^^,^^96^ Adding arginine-rich peptides to PMOs restored muscle and cardiac dystrophin expression in the *mdx* mice by exon 23 skipping.^95^^,^^97^^,^^98^ Interestingly, in a recent pre-clinical study, in which the PPMO antisense oligo was applied to hDMDdel52/*mdx* mice for exclusion of exon 51, quite high efficacy of dystrophin restoration in the heart, reaching 7.7% versus 0.5% in saline-treated animals, was observed.^99^

At least three PPMOs have been evaluated in clinical trials: vesleteplirsen (SRP-5051) conjugated to a PMO directed toward exon 51 (Sarepta Therapeutics, ClinicalTrials.gov: NCT04004065, NCT03375255); PGN-EDO51, developed by PepGen, a PMO targeting exon 51 connected with a linear peptide (ClinicalTrials.gov, NCT06079736); and ENTR-601-044, an Entrada Therapeutics’ PMO binding to exon 44 with cyclic peptide (ISRCTN36174912). However, both in the nonhuman primate studies and in DMD patients, hypomagnesemia has been reported, suggesting a kidney dysfunction and possible PPMO toxicity (reviewed by Aartsma-Rus^100^). Based on these findings, on November 6, 2024, Sarepta Therapeutics announced the discontinuation of PPMO development.^101^ In addition to creating new PMOs by adding, for example, peptides, attempts have been made to improve their specific delivery to skeletal muscle by conjugating antibodies targeting transferrin receptor 1 (TfR1), expressed on the surface of skeletal, smooth, and cardiac muscle cells, among others.^102^ EXPLORE44 (ClinicalTrials.gov: NCT05670730), a randomized, placebo-controlled, double-blind phase 1/2 trial, tests a full transferrin 1 antibody conjugated to PMOs targeting exon 44 (AOC 1044, Avidity Biosciences).^103^ In another study (ClinicalTrials.gov: NCT06280209), chemically modified, negatively charged BMN351, generated by BioMarin Pharmaceuticals, will be checked in patients with genetic mutations amenable to exon 51 skipping. Advancements in ASO-based therapy have prompted the suggestion of using a cocktail of various ASOs (multi-exon skipping) to more effectively restore the dystrophin mRNA open reading frame. Such a strategy might apply to 80%–90% of DMD patients, regardless of mutation type.^104^^,^^105^ This interesting approach for treating DMD was first tested in the canine X-linked muscular dystrophy (CXMD) dog model, harboring a splice site mutation in intron 6, leading to a lack of exon 7 in dystrophin mRNA. The multi-exon skipping of exons 6 and 8 led to the correction of the reading frame and resulted in the truncated dystrophin expression in canine skeletal muscles.^106^ Further studies showed that the PPMOs cocktail designed to skip dystrophin exons 6 and 8 after four systemic administrations into CXMD dogs rescued dystrophin expression in the myocardium and cardiac Purkinje fibers and improved cardiac conduction abnormalities in the dystrophic heart.^107^ These results indicate the effective applicability of ASOs in both muscle and cardiac DMD dysfunctions, which can hopefully be applied as a routine treatment of DMD patients in the future.

As indicated, ASO-based exon skipping is highly personalized, since specific ASOs can be applied only to patients with particular mutations. Moreover, it is not a one-time treatment, as current forms of ASO require weekly injections. Accordingly, recent analyses raise questions not only about the medical effectiveness of antisense therapies in DMD but also about their economic burden. For example, Eteplirsen was introduced at an annual cost of approximately $300,000 (USD), yet real-world data indicate that nearly one-third of patients discontinued therapy after a median of 7 months due to insufficient perceived benefit.^108^ Despite concerns about the true effectiveness of ASOs, it should be noted that initial clinical trials primarily enrolled patients in the early stages of DMD (mean age 8.5 ± 2.0 years), whereas post-approval treatment has mostly been administered to patients at more advanced stages of the disease (mean age 13.7 years). Of note, these patients predominated among those who discontinued the therapy.^108^ According to Bendicksen et al.,^75^ Sarepta Therapeutics reported $612 million in net revenue from ASO products in 2021. Meanwhile, there is still a lack of convincing evidence for the clinical benefits of ASOs for DMD patients.

Even if successful, restoration of dystrophin by exon skipping can face other obstacles. For example, miR-146a, an inflammation-related microRNA, is increased in DMD.^109^^,^^110^ Therefore, there is a risk that miR-146a can target the 3′UTR of dystrophin mRNA, potentially diminishing the effects of exon skipping. Accordingly, it has been demonstrated that the deletion of miR-146a enhances dystrophin restoration in the exon 52-deleted *mdx52* mice.^111^ This aspect needs to be thoroughly studied in the future.

Other investigated approaches combine exon skipping with gene therapy by harnessing a U7 small nuclear ribonucleoprotein (snRNP) cassette engineered to induce exon skipping.^3^^,^^112^ This has been applied directly *in vivo* by AAV-mediated delivery^113^ or *ex vivo* by stable (e.g., lentiviral) modification of muscle progenitor cells.^114^ Additionally, a strategy has been explored that exploits the potential of using an IRES located in exon 5 to initiate translation of the dystrophin mRNA downstream of the canonical cap site and a frameshifting mutation, specifically in the case of exon 2 duplications.^115^ Finally, exon skipping can also be achieved by CRISPR/Cas editing, which, in contrast to ASOs, creates the chance for a permanent effect and could be performed only once (discussed below).

### Gene therapy—restoration of dystrophin expression

As mentioned, the full-length dystrophin mRNA coding sequence is 11.2 kb, exceeding the carrying capacity of most viral vectors. Therefore, non-viral delivery methods displaying several potential advantages over viral vector technology, including the potential for re-administration, higher packaging capacity, and easier large-scale production, have been tested. However, early attempts at intramuscular plasmid-based delivery of full-length dystrophin resulted in minimal expression at the injection sites, and naked plasmids are unlikely to be systemically delivered for bodywide muscle targeting.^116^ The development of helper-dependent “gutless” adenoviral vectors (HD-AdV) raised hope for their use in DMD treatment; however, the preclinical results^117^^,^^118^ have not been translated. The high immunogenicity of HD-AdV and difficulties in their production, combined with an extremely high tropism for the liver, create significant barriers. Counsell et al.^119^ successfully used lentiviral vectors to deliver full-length dystrophin to DMD cells. However, conventional lentiviral vectors are not good candidates for *in vivo* delivery, and their application could be associated with the risk of insertional mutagenesis. Interestingly, a newer generation lentiviral vector displaying myogenic tropism was recently described that can target dystrophic skeletal muscles bodywide and can be administered repeatedly, raising the prospect of an alternate delivery system for mini-dystrophins.^120^

AAV vectors are currently the most widely used and effective system for skeletal and cardiac muscle-specific gene therapy, due to their natural tropism to these tissues and their ability to extravasate from the vasculature, enabling systemic delivery.^68^ Recently, AAV vectors with improved muscle tropism have been generated by directed evolution or DNA shuffling.^121^^,^^122^ However, the cargo capacity of AAVs is limited, allowing only ∼4.7 kb of DNA to be delivered. Therefore, many shorter, rod-truncated versions of the dystrophin gene lacking the C-terminal domain were created to overcome the limitations related to transgene size.^70^

**Figure 3:**
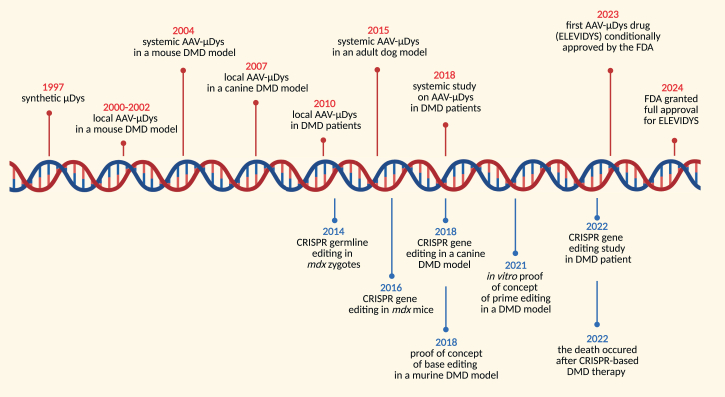
Progress in gene-targeted therapeutic strategies in DMD The history of studies with AAV-based microdystrophin (μDys) (upper part) and CRISPR/Cas9-mediated correction of *DMD* (lower part) in animal models and DMD patients.

**Figure 4:**
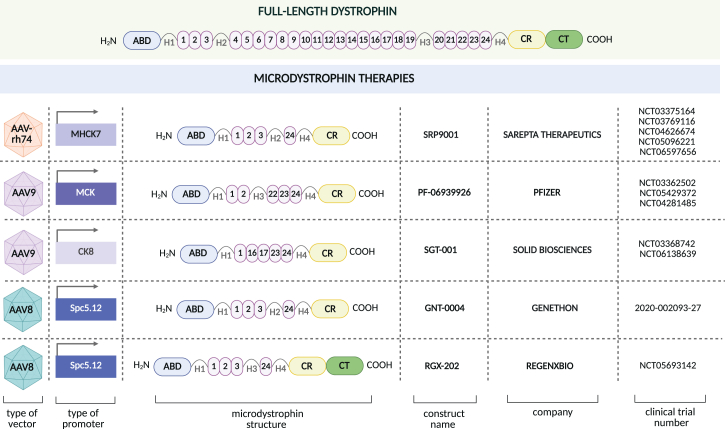
Microdystrophin therapies for DMD Several companies are developing various microdystrophin (μDys) constructs, lacking specific domains of full-length dystrophin, delivered using different types of adeno-associated viral (AAV) vectors. The numbers of clinical trials are shown (based on Clinicaltrials.gov; for GNT-0004, the EudraCT number is provided).

Several small dystrophins that can be carried by AAV vectors have been tested experimentally in several clinical trials^73^^,^^123^^,^^124^^,^^125^^,^^126^ (Table S3; Figures 3 and 4). Despite the confusing use of alternate names “miniDys” or “microDys” (μDys), all such clones are of similar sizes and carry either four or five of the spectrin-like repeats in the dystrophin central rod domain (RD). In 2004, we (Chamberlain’s team) demonstrated that systemic delivery of a μDys using AAV6 resulted in robust μDys expression in murine skeletal muscles and heart.^68^ This study provided the proof of principle for the application of AAV vectors to the treatment of DMD. Importantly, in follow-up studies, we also showed that AAV6-μDys significantly extends the life span of severely affected dystrophin/utrophin double knockout mice (dKO). While 80% of untreated dKO mice die within 15 weeks, systemic delivery of 3 × 10^12^ AAV-μDys vectors to 1-month-old dKO mice led to uniform dystrophin expression for at least one year and prolonged mouse survival.^124^ Notably, treatment of 20-month-old *mdx* mice with AAV6-μDys also resulted in amelioration of dystrophic phenotype.^68^

Numerous follow-up studies also demonstrated a significant effect of AAV-μDys expression in ameliorating the DMD phenotype in mice. An important advancement was the design of the muscle-specific CK8e and MHCK7 promoters, both synthetic derivatives of the muscle CK enhancer and promoter elements, which provide robust expression restricted to skeletal and cardiac muscles.^125^^,^^127^ AAV-mediated μDys expression was also effective in DMD dogs.^128^^,^^129^ Le Guiner et al.^130^ demonstrated long-term expression using the AAV2/8 serotype, while Birch et al.^131^ showed improved strength through the systemic delivery of AAV9-CK8e-μDys. Howard et al.^132^ showed that μDys gene therapy ameliorated cardiomyopathy in Fiona DMD mice. These animals lack dystrophin and utrophin expression in the heart; however, partial expression of utrophin in skeletal muscles protects them from the premature death typically seen in global dKO *mdx*/utrophin animals. Treatment of Fiona mice at 4 weeks of age prevented the development of heart failure, as analyzed 12^132^ and 18 months later.^133^

In humans, the intravenous infusion of μDys constructs utilizing AAV8, AAV9, or AAVrh74 has been tested (Table S3). Recombinant AAV serotype rh74 (rAAVrh74) is a vector isolated from rhesus monkeys and is claimed to display lower immunogenicity than other serotypes isolated from humans (e.g., AAV2, AAV5, and AAV9) and high muscle transduction efficiency, although recent clinical data cast doubt on this assertion (see below).^134^ The rh74 vector was combined with an MHCK7 cassette driving expression of the very first μDys clone developed^70^^,^^127^ to generate delandistrogene moxeparvovec (Elevidys). The initial clinical studies of this vector were performed in Study 101 (NCT03375164), Study 102 (NCT03769116), and Study 103 (ENDEAVOR; NCT04626674) sponsored by Sarepta Therapeutics (Table S3; Figures 3 and 4). Recently, the outcome from the first part of the EMBARK phase 3 trial was released. Mendel et al.^135^ demonstrated that the primary endpoint change from baseline in the North Star Ambulatory Assessment (NSAA) score did not reach statistical significance between DMD patients after a single intravenous administration of delandistrogene moxeparvovec (1.33 × 10^14^ vector genomes per kilogram of body weight; vg/kg; *n* = 63) and the placebo group (*n* = 62). Although some improvement was found in younger boys between 4 and 5 years of age, no (statistically) significant treatment effect was detected in older DMD individuals aged 6–7 years. At week 52, key secondary endpoints (time to rise from the floor and 10-meter walk and/or run) were also not meaningfully changed. Although patients treated with Elevidys showed a 0.64-s shorter time to rise from the floor and a 0.42-s shorter time for a 10-meter walk, the biological significance of these differences is disputed.

Despite all uncertainties, in June 2024, the US FDA granted full approval for delandistrogene moxeparvovec for ambulatory patients with DMD aged 4 years and older. This decision was based on data demonstrating the expression of μDys after treatment, e.g., mean expression detected by western blot at week 12 was 34.29% in the treated group compared to 0% in the placebo group.^135^ The accelerated, conditional approval was also granted for non-ambulatory patients (however, see below). Interestingly, recent preclinical studies in *DMD*^MDX^ rats showed that systemic delivery of delandistrogene moxeparvovec (1.33 × 10^14^ vg/kg) over 52 weeks more than doubled median survival (from 13 to over 25 months) and produced substantial improvements in cardiac function.^136^ These findings may reflect species-specific differences in the response to treatment, underlining the need for further research to fully understand efficacy, optimize dosing, and evaluate long-term outcomes in humans.

Recently, so-called MYOAAV and AAVMyo vectors, which exhibit increased muscle tropism, have been identified through muscle-directed high-throughput screening (HTS).^122^^,^^137^^,^^138^ Of note, these myotropic vectors are characterized by the presence of the common amino acid motif RGD (arginine-glycine-aspartate) on their capsid surface, which is thought to mediate targeting to the integrin complex. Integrins, expressed on muscle and endothelial cells, are commonly exploited by viruses through the RGD motif, facilitating cell-specific attachment and entry.

This finding provides the foundation for the RGD-dependent integrin-targeting AAV-specific myotransduction, which is reflected by high effectiveness in correcting dystrophic phenotypes in DMD mouse models, even at relatively low doses, ranging from 2.0 × 10^12^ vg/kg to 1.0 × 10^13^ vg/kg. Additionally, a new vector type, combining binding motifs of human integrin alphaV beta6 with a liver-detargeting capsid, was recently proposed by Vu Hong et al.^139^ This vector was superior to AAV9 in delivering μDys *in vivo* while liver targeting was reduced. The first such myotropic AAV vector to be used in humans (containing AAV-SLB101 capsid targeting integrin receptors) has begun testing in a DMD clinical trial (INSPIRE DUCHENNE, ClinicalTrials.gov, NCT06138639), and a recent report showed widespread and high expression of microdystrophin in the vast majority of myofibers in the sampled skeletal muscles.^140^

### Safety issue of AAV-μDys gene therapy

Regardless of the type of μDys and AAV vector used, the therapy is associated with several challenges and limitations. A key issue is the potential activation of immune responses. These can be triggered by the AAV capsid proteins, unmethylated CpGs in the vector DNA, or the therapeutic μDys protein.

It has been demonstrated that a cytotoxic T lymphocyte immune response may play a crucial role in serious adverse events such as liver toxicity, myositis, and myocarditis. Moreover, the humoral immune response, i.e., the presence of antibodies against AAVs, poses an additional significant challenge to AAV-mediated gene therapy, as antibodies induced by AAV administration currently prevent the possibility of their re-dosing. It should be emphasized that the primary focus in DMD therapy, meaning targeting striated muscle, which constitutes more than 40% of body mass, requires extremely high doses of vectors infused intravenously (≥1.0 × 10^14^ vg/kg for conventional AAV serotypes) to achieve substantial therapeutic efficacy.^141^ Predictably, administering such high vector doses appears to be correlated with increased immunogenicity, leading to hepatotoxicity (as the AAV vectors preferentially accumulate in the liver), thrombotic microangiopathy (TMA), and atypical hemolytic uremic syndrome (aHUS) (reviewed by Kishimoto & Samulski^142^; see also ref.^143^).

Overall, DMD patients’ conditions may predispose them to the side effects of gene therapies, as older patients have aggravated muscle degeneration and inflammation (for more information about the adverse effects of DMD gene therapy, see review by Duan^144^). Screening for anti-AAV antibodies and capsid-specific T cells can help identify patients for whom gene therapy with a particular AAV serotype may pose challenges. Consequently, gene therapies could prove safer and more efficacious in the youngest patients, although even very young DMD boys display hallmarks of muscle damage.

It has become clear that a specific type of DMD mutation (exon 8–11 deletions; this region encodes the very end of the N-actin binding domain, H1, and the beginning of the first spectrin-like repeat) confers significant risk of developing an immune response against μDys.^145^ Serious cases of myositis and myocarditis observed in five patients who received μDys gene therapy in various trials were associated with different AAV serotypes (AAV8, AAV9, and AAVrh74). These patients were injected with AAV-μDys carrying various muscle-specific promoters (Spc5.12 or MHCK7) and at different doses (ranging from 1 × 10^13^ to 2 × 10^14^ vg/kg). This prompted the suggestion that the reactions were due to an immune response against dystrophin epitopes encoded by the transgene. Indeed, all five patients had large deletions from exon 8 to exon 21, while the epitopes encoded by exons 8 through 11 were present in all AAV constructs used in these clinical trials. Therefore, patients with similar genomic deletions (exons 8–11) are now being excluded from treatment with these vectors. Importantly, such an agreement was reached through the pre-competitive collaboration among the companies sponsoring the clinical trials.^145^

Therefore, attempts are made to generate new μDys constructs to minimize dystrophin-specific T cell responses and increase muscle and heart protection. Wasala et al.^146^ noted that all μDys constructs tested in clinical trials carry H1 and H4. To dissect their roles, genetically engineered constructs lacking H1 (ΔH1) or H4 (ΔH4) were compared with H1/H4 (containing both H1 and H4). When delivered in AAV9 to *mdx* mice, constructs possessing H4 were superior to ΔH4, suggesting that H4 is essential for μDys function. Indeed, hinge 4 carries a WW domain, which is important for binding to β-dystroglycan.^147^ In contrast, H1 enhances dystrophin function but is not absolutely needed.^146^ Specifically, H1 deletion did not affect μDys subcellular localization, DGC restoration, or ECG improvement but reduced muscle function in force assays. These findings provide valuable insights for the development of next-generation μDys therapies with improved functional outcomes while potentially minimizing immunogenicity. Given that H1 may carry a dominant immunogenic epitope associated with severe adverse events in clinical trials (as discussed above), its removal from μDys could attenuate transgene-triggered immune responses. Nevertheless, the effects of this modification on the overall function and therapeutic efficacy of the construct suggest that alternate sequence modifications may be important for reduced immunogenicity without significant loss of function.

Recently, Verma et al.^148^ screened 101 DMD patients for neutralizing and binding antibodies against various AAVs. Binding antibodies were detected in nearly all DMD individuals, while the presence of neutralizing antibodies was confirmed in ∼30%–50% of patients and was dependent on the AAV serotype. The lower seroprevalence for AAVrh74 (32%) and AAV9 (36%) suggested their superiority for wider use as vectors for gene therapy compared to AAV8 and AAV2, which were detected in 47% and 56% of DMD males, respectively. Currently, before treatment with AAV-μDys, DMD patients are screened for neutralizing antibodies against AAVs, and if high levels are detected, they are excluded from trials (e.g., AAV9 in Pfizer’s trials) (Table S3).

Additionally, although pre-existing dystrophin-specific T cell responses were observed in a smaller number of DMD individuals,^149^^,^^150^ these findings should be considered before proceeding with clinical trials and future studies on DMD. Accordingly, Sarepta Therapeutics plans to conduct a phase 1 interventional study (NCT06597656) in DMD patients with pre-existing antibodies to AAVrh74. The study aims to evaluate the feasibility of removing these antibodies before administering the gene transfer through therapeutic plasma exchange (plasmapheresis). Importantly, experiments performed in non-human primates (Chinese rhesus macaques) demonstrated the safety of such a procedure.^151^ Plasmapheresis, when performed before redosing of SRP-9001, resulted in reduced levels of circulating antibodies to AAVrh74. This finding offers hope for multiple administrations (redosing) of viral vectors in patients or the application of a new form of AAV-based therapy in the future. Additionally, multiple ongoing studies are testing if and how various immunosuppressive regimens might affect gene therapy and humoral or cell-mediated immune responses.

Other serious adverse effects, including thrombotic microangiopathy (TMA) and renal failure linked with complement activation, were reported to occur in patients receiving AAV doses greater than 2.0 × 10^14^ vg/kg (https://www.fiercebiotech.com/biotech/solid-bio-safely-doses-dmd-gene-therapy-after-exiting-fda-hold and https://www.pfizer.com/news/press-release/press-release-detail/pfizers-new-phase-1b-results-gene-therapy-ambulatory-boys). Recently, Byrne et al.^152^ reported detailed evidence of TMA in three out of 22 participants in the NCT03362502 clinical trial, conducted by Pfizer. This occurred within 2 weeks after infusion with rAAV9-based therapy for fordadistrogene movaparvovec (3 × 10^14^ vg/kg). Notably, all three participants had negative genetic testing for susceptibility to TMA. In these patients, strong type 1 interferon (IFN) response was observed within 2–4 days post-infusion, followed by a rise in immunoglobulin G (IgG) and IgM anti-AAV9 antibodies by day 7 and complement activation (C3/C4 depletion, C5b-9 increase) by days 4–8. Fortunately, TMA could be successfully managed. The type I IFN response can be modulated using glucocorticoids, Toll-like receptor (TLR) inhibitors, or JAK-STAT pathway blockers; immunosuppressants such as sirolimus, cyclosporin, and rituximab target IgM and IgG production, while complement activation can be inhibited by eculizumab, the C5 complement inhibitor (reviewed by Costa-Verdera^153^). Nevertheless, careful monitoring during the early period following AAV administration remains essential. Most recently, two older, non-ambulatory DMD boys treated with Elevidys died of acute liver failure approximately 2 months after dosing (https://www.statnews.com/2025/06/15/duchenne-sarepta-gene-therapy-elevidys-patient-death/). While the mechanism behind this liver failure has not yet been identified, Sarepta Therapeutics has halted further enrollment of non-ambulatory patients in their trials. Together, these collective serious adverse events and deaths associated with high-dose AAV delivery point to a need for improved vectors and delivery strategies.^143^

Additional safety issues are expected to arise in clinical trials of genetic therapies based on CRISPR/Cas gene editing following delivery by AAV. In such cases, besides restoring dystrophin and delivering AAV antigens, an immune response is likely to develop against the bacterial Cas enzymes.

### Further pre-clinical development of dystrophin gene therapy

**Figure 5:**
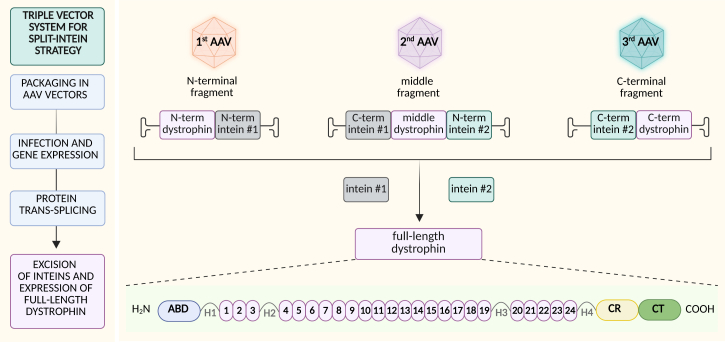
Triple vector system for split-intein approach in DMD Full-length dystrophin is divided into three fragments, linked to two pairs of split inteins, and delivered separately into cells. Upon expression, the inteins mediate the ligation of the dystrophin fragments into a functional, full-length dystrophin protein.

μDys proteins are not fully functional (e.g., they are only one-third the size of full-length dystrophin) and, as such, may not provide sufficient therapeutic benefit. However, recent studies demonstrate that AAV vectors can facilitate the expression of larger and full-length dystrophins. The ability of two or more AAVs to recombine after delivery to the same cell type has been previously exploited.^154^^,^^155^ This can be achieved by homologous recombination of AAV DNA or the formation of head-to-tail AAV concatemers and *trans*-splicing of the pre-mRNA across the ITR junction. These processes allow the reassembly of partial DMD sequences inside the cells. Despite their potential, these approaches are not highly efficient,^156^ and unwanted products from vector concatamerization could be formed.^155^ Recently, novel strategies, relying on the split-intein approach, have provided a means to express full-length dystrophin. Inteins are small bacterial polypeptides that self-splice after translation (a process known as protein *trans*-splicing—PTS). For AAV applications, particularly suitable are split inteins, which can be expressed as two separate peptide fragments; thus, they can be expressed from two different AAV vectors. Once inside the cell, these fragments associate and splice out, enabling the joining of two separate protein fragments (exteins) into a single, functional protein. This mechanism allows the N-terminal part of dystrophin to be joined with the middle part, and then the C-part, when three AAV vectors harboring different parts of DMD are expressed in the same cell (Figure 5). However, while one such dual vector approach had been previously tested in *mdx* mice, the designed vectors did not provide significant dystrophin expression or functional improvements after intramuscular administration.^157^ By significantly refining and enhancing this approach, we (Chamberlain’s team) tested different types of split-inteins and strategic design of their placement within dystrophin to select clones that facilitated efficient and functional dystrophin expression.^158^ The studies demonstrated that both midiDys (delivered with two AAV vectors) and full-length dystrophin (delivered by three AAV vectors) can be expressed in the skeletal muscles and the hearts. One such midiDys construct carried the entire N-terminal actin binding, 13 properly phased SRs, all four hinge regions, as well as the CR and CT domains. Importantly, such a construct is larger and more functional than a protein expressed in some BMD patients carrying a deletion of 46% of the DMD gene, who were largely asymptomatic and ambulant past their 60s.^159^ The approach has been initially tested in young, 8-week-old *mdx*^4cv^ mice.^158^ Three months after systemic delivery of AAV6 vectors at the total dose of 2 × 10^14^ vg/kg, dystrophin expression reached 75% of wild-type level in tibialis anterior for midiDys (two AAV vectors), about 30% for full dystrophin (three vectors), but only 8% for μDys (one vector). Interestingly, the level was higher in the heart, reaching about 65% for μDys, 62% for full dystrophin, and 178% for midiDys. Importantly, these effects, independent of the number of vectors used, were achieved with the total dose of the vectors not exceeding 2 × 10^14^ vg/kg.^158^ To enhance this approach, an AAV9 vector harboring μDys was compared with AAVMyo-based delivery of μDys or midiDys (two vectors), under the control of the CK8e-muscle-specific promoter. Of note, midiDys at a low dose (2 × 10^13^ vg/kg) improved tibialis anterior mechanical properties, while this was not achieved with low-dose μDys delivery. Importantly, the high dose of single or two AAVMyo-dystrophin constructs resulted in the normalization of CK levels, a serum marker of DMD. In a follow-up study,^160^ we compared the effectiveness of full-length dystrophin delivery via co-delivery of three myotropic AAV vectors in young and old *mdx*^4cv^ mice. Eight-week-old or 17-month-old mice were systemically administered either 4 × 10^13^ or 8 × 10^13^ vg/kg (total) vectors at a 1:1:1 ratio of split N-terminal, middle, and CT intein/dystrophin constructs. We demonstrated that the triple vector approach significantly corrected muscle defects and restored normal force in both hindlimb and diaphragm muscles when given early in DMD progression. At later stages, when given to 17-month-old mice, it remains effective in hindlimb and cardiac muscles but provides limited benefit for the diaphragm, which by this age has lost significant muscle mass. This study highlights the effectiveness of combining low doses of myotropic AAVs with effective split inteins in driving the expression of full-length dystrophin and functional improvements, even in severely affected animals.^160^

Finally, a significant amelioration of the DMD phenotype has also been noted in 24-month-old animals that had already developed heart failure. Besides an improvement in heart function, an increase in skeletal muscle force was also achieved in these old mice. Importantly, AAVMyo vectors achieved comparable dystrophin expression and functional effects at a lower dose (10^13^ vg/kg) compared to AAV9 vectors, which were ineffective at this dosage. When applied in 17-month-old mice, the effect persisted until they reached two years of age, and a significant decrease in skeletal and cardiac muscle fibrosis was observed after dual AAVMyo midiDys treatment. Of note, in these old dystrophic animals, only dual-vector-mediated midiDys expression, but not μDys, was protective, which may indicate enhanced stability and function of midiDys in older animals.

Another group has tested a similar approach.^161^ The intein-based strategy was effective with the MyoAAV4 vector, another myotropic serotype.^121^ In the same *mdx*^4cv^ DMD model, significant expression of full-length dystrophin was achieved, reaching 62% of the wild-type levels in the heart and 75% in skeletal muscle, in 9- to 10-week-old dystrophic mice examined 6 weeks after treatment.^161^ These experiments demonstrated the comparable effectiveness of full-length dystrophin and μDys delivered with MyoAAV4 at the dose of 8 × 10^13^ vg/kg. Interestingly, only full-length dystrophin restored the membrane localization of cavin-4 in cardiomyocytes, leading to improved ERK phosphorylation. Moreover, the study showed that MyoAAV4 vectors provided a more robust expression of μDys than full-length dystrophin, reaching 2.6- to 3.7-fold and 30%–50% of the wild-type level, respectively. However, as the functional effects were similar, the authors conclude that even lower restoration of full-length dystrophin may provide similar protection as overexpressed μDys.

Split-intein approaches are also being tested in other genetic strategies that require AAV vectors to deliver long transgenes. In the case of DMD, such vectors are applied to express CRISPR/Cas editing systems (discussed below). The strategy holds promise and may effectively address the challenge of expressing functional, non-truncated proteins. However, it also generates several obstacles that need to be addressed before initiating clinical trials. Although inteins are removed during splicing, they could provoke an immune response due to their size (125–150 amino acids) and the uncertainty regarding their intracellular half-life. Moreover, despite splicing, residual intein footprints consisting of up to six amino acids can remain in the reconstituted protein. These residues are critical for catalyzing the final ligation of proteins and are derived from the last and first tripeptide sequences of the N- and CT halves of the extein. Besides triggering an immune response, such a “footprint” could affect the folding and function of the therapeutic protein. Finally, as μDys itself was also demonstrated to be immunogenic in some patients (see discussion above), it remains to be determined whether the combination of both dystrophin and (the rest of) intein may be a stronger immune stimulant than dystrophin alone. Such immune responses have not been seen in rodents, but are being examined in additional models. Fortunately, numerous inteins are available for testing, as described.^158^

## Gene editing

All the strategies discussed above have inherent limitations. ASO-based exon-skipping approaches restore shortened dystrophin expression with modest efficacy. Moreover, they must be dosed continuously due to the short lifespan of ASOs and their effect on DMD pre-mRNA. Finally, these therapies are only applicable to patients with specific types of mutations. Gene therapy based on AAV transfer of μDys delivers the sequence encoding a shorter, hence only partially functional, protein. This can be overcome by using AAV split-intein constructs to deliver a full dystrophin sequence.^158^ However, again, the efficacy of registered AAV-μDys therapy remains limited, and its long-term effects are still unknown. The development of CRISPR/Cas-based gene editing strategies offers an alternate approach, though they share some of the same limitations as the previous strategies. The main advantage over ASO-based exon skipping is that CRISPR/Cas-based editing occurs directly at the genome level, offering the possibility of a one-time treatment with a long-term effect, although tailored correction for specific mutations is still required.

**Figure 6:**
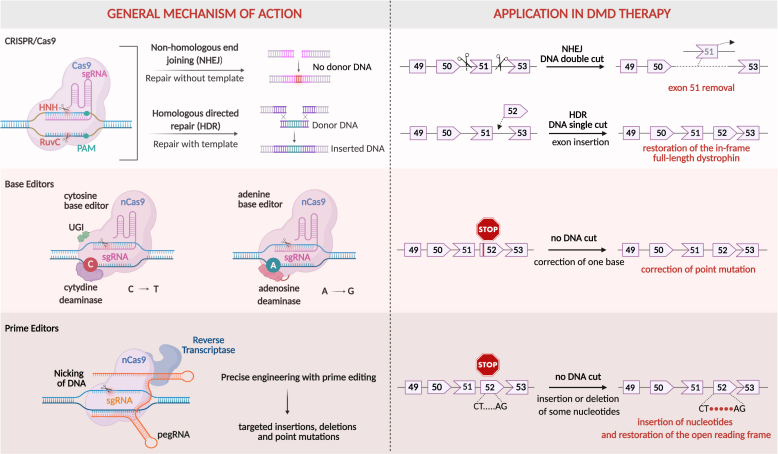
Genome-editing technologies: CRISPR/Cas9, base editors, and prime editors and their possible application for *DMD* repair The CRISPR/Cas9 system relies on the ability of CRISPR guide RNAs (gRNAs) to direct the Cas9 endonuclease to specific genomic sequences, where Cas9 induces a double-strand break (DSB). The break is repaired through nonspecific non-homologous end joining (NHEJ) or homology-directed repair (HDR) mechanisms (upper part). Base editors using catalytically inactive Cas9 (Cas9 nickase) facilitate the direct conversion of one DNA base to another (cytosine deaminase [CBE] converts cytidine to thymidine, and adenosine deaminase replaces adenosine to guanine) without inducing DSBs (middle part). Prime editors employ a longer guide RNA, known as prime editing gRNA (pegRNA), along with a reverse transcriptase enzyme, guided by dCas9, to directly replace small DNA sequences at target loci (lower part).

The classical gene editing method utilizes the Cas9 nuclease, which is targeted near the site of the *DMD* mutation. By causing a double-strand DNA break, Cas9 initiates repair mechanisms that can correct the mutation and induce alternative splicing (Figure 6). Specific targeting of Cas9 is preserved by the single guide RNA (sgRNA), which directs the Cas9 to the region containing a particular sequence of nucleotides known as the protospacer adjacent motif (PAM). The PAM sequence is 5′NGG nucleotides for the *Streptococcus pyogenes* Cas9 (SpCas9) and 5′NNGGRT for the *Staphylococcus aureus* Cas9 (SaCas9), the two most commonly used nucleases. Cas9, through its RuvC and HNH nuclease domains, cuts both DNA strands three nucleotides upstream of the PAM sequence. This double-strand break initiates DNA repair mechanisms, primarily through nonspecific non-homologous end joining (NHEJ) or homology-directed repair (HDR). NHEJ is a nonspecific process, resulting in the generation of short indels (deletions or insertions), while HDR uses a homologous template to replace the damaged DNA region. The latter is most efficient in dividing cells; hence, in post-mitotic skeletal muscles and cardiomyocytes, the DNA breaks are mostly repaired by NHEJ. In the context of DMD, if Cas9 is targeted to the sequences flanking an exon adjacent to the mutation disrupting the reading frame, the repair mechanism can mutate (or repair) a splice donor or acceptor, leading to exon skipping and restoration of an open reading frame. Accordingly, the HDR approach can be applied to correct the mutation in the *DMD* exon(s) by replacing it (or several of them) using a homologous repair template containing the correct DMD sequence delivered alongside Cas9 and sgRNA. However, the efficiency of HDR-mediated correction of the DMD mutation in skeletal muscles and the heart is low. Despite this limitation, this strategy successfully corrected the point mutation in exon 23 of *mdx* mouse zygotes.^162^

In recent years, novel approaches have been developed, in which the targeting property of modified Cas9 (partially or fully devoid of nuclease activity) has been exploited for the specific correction of point mutations (base editing [BE] and prime editing), activation of gene expression by transcriptional targeting of the promoters, or epigenetic modifications.

BE utilizes a catalytically inactive version of Cas9, known as “dead” Cas9 (dCas9), or a Cas9 variant with a mutated HNH domain. This approach retains the nickase activity of Cas9 and combines it with deaminase enzymes to enable precise single-base changes in DNA. Initially, the cytosine base editor (CBE) was introduced, which converts a C-G base pair to a T-A base pair. This occurs through the deamination of cytidine to uridine, which is subsequently recognized by the cell’s DNA polymerase as thymidine, pairing with adenine on the complementary strand. Since eukaryotic adenine deaminases do not exist naturally, an adenine base editor (ABE) was engineered from *Escherichia coli* TadA, a tRNA adenosine deaminase. ABE deaminates adenine, converting an A-T base pair into a G-C base pair (Figure 6).

While BE can correct only 6 out of 12 possible point mutations, prime editing (PE) can potentially correct all types of mutations (reviewed by Chen and Liu^163^). In PE, a nickase SpCas9 (nCas9) is fused to the engineered reverse transcriptase (commonly from murine Moloney leukemia virus) and combined with the prime editing guide RNA (pegRNA), which consists of three parts: sgRNA (from 5 to 3′) that anneals to the desired target site, a scaffold for nCas9, and the correction template (RT template) that contains the desired, correct sequence, together with a primer binding site that binds to the non-target DNA strand. Reverse transcriptase uses this pegRNA to introduce the desired genetic changes, such as correcting point mutations or introducing short insertions or deletions. To date, traditional PE has been effective mainly for correcting short DNA segments. However, several modifications of this system, such as twinPE combined with integrases or the PASTE (programmable addition via site-specific targeting elements) system, have been successfully implemented to enable the integration of larger cargos, up to ∼40 kb (reviewed by Chen and Liu^163^).

### Application of CRISPR/Cas9 for research and therapy of DMD

The capacity of CRISPR/Cas9 to introduce mutations has been harnessed to establish novel models of DMD. This includes the generation of isogenic iPSC lines (subsequently differentiated into cardiomyocytes or myoblasts), where specific mutations that mimic those occurring in patients can be introduced^164^ (reviewed by Chemello et al.^165^). A significant advantage of CRISPR/Cas9 is its capacity to accelerate the generation of new animal models of disease. Accordingly, several DMD animals have been established by deletion of specific exons, including mice_,_^166^^,^^167^ rats,^168^ rabbits,^169^ pigs,^170^^,^^171^^,^^172^ and rhesus monkeys.^173^ Notably, a multi-exon deletion of exons 52–54 allowed the generation of a novel *Dmd* Δ52-54 mouse model, which exhibits early onset hypertrophic cardiomyopathy.^174^ Additionally, it has been demonstrated that base editors can introduce specific mutations into murine zygotes, generating dystrophic mice.^175^

The first proof-of-principle study for therapeutic CRISPR/Cas editing was performed by Eric Olson’s group in 2014, demonstrating that CRISPR/Cas9 could correct the *Dmd* mutation in *mdx* mice^162^ (Table S4). Germline editing of the exon 23 mutation generated mosaic animals, demonstrating correction in 2%–100% of the *Dmd* gene. This correction has been achieved through the NHEJ, but an even better outcome was observed when the repair template was injected into the zygote together with Cas9 protein and sgRNA, resulting in mutation correction by HDR.^162^ Similarly, the same group utilized the *Lachnospiraceae bacterium* Cpf1 nuclease, where mRNA, sgRNA, and repair template were injected into the *mdx* zygote, leading to dystrophin correction through both NHEJ and HDR.^176^

Three seminal studies^138^^,^^177^^,^^178^ were the first to demonstrate the feasibility of correcting the mutation in newborn mice. They utilized the AAV9 vector and achieved restoration of the reading frame in a significant percentage of the dystrophin mRNAs in *mdx* mice through the excision of exon 23 (Table S4). Next, we (Chamberlain’s team) successfully corrected the mutation in exon 53 of *mdx*^4cv^ mice.^179^ This editing involved the removal of the 45 kb region, encompassing exons 52 and 53, which restored the reading frame by connecting exons 51 to 54 in the pre-mRNA.^179^ Numerous subsequent experiments have further validated the feasibility of this approach (Table S4). For example, using SaCas9, El Refaey et al.^180^ corrected the *Dmd* mutation in exon 23 of *mdx*/*utrn* ^±^ mice, reporting a 40% restoration of dystrophin expression in cardiac muscles. In this study, the authors also confirmed that systemic AAV delivery of the larger SpCas9 could correct the mutation. Although SaCas9 was slightly less efficient than SpCas9, the editing resulted in a reduction of cardiac fibrosis and an improvement in contractility.

CRISPR/Cas9 was used not only to correct DMD mutations but also to generate them. Amoassi et al.^166^ applied both strategies. First, they generated a mouse model of DMD by deleting exon 50. In the next step, they used the AAV approach to deliver the editing system, which deleted exon 51, restored the *Dmd* reading frame, and achieved up to 90% dystrophin expression in skeletal muscle and the heart.^166^ Similarly, Olson’s group also created a new *mdx* strain by deleting exon 44 and then restoring a truncated dystrophin expression by removing exon 45.^181^ This was also achieved when the AAV9-expressing gene-editing components were delivered systemically.

Hakim et al.^182^ showed that administering a sufficient dose of sgRNA and AAV-Cas9, designed to excise exon 23, to 6-week-old *mdx* mice resulted in long-term correction of most striated muscles, including the heart. Importantly, dystrophin expression lasted for up to 18 months, leading to improved skeletal muscle function, better cardiac hemodynamics, and a reduction of fibrosis across all muscles. Nelson et al.^183^ also reported the persistence of the effect of such a strategy in *mdx* mice for 12 months. The important observation from this study was that AAV-Cas9 was immunogenic when delivered into adult mice; however, this could be overcome by treating neonatal animals.^183^ A long-term effect of AAV-SaCas9-gRNA targeting introns 20 and 23 to restore the reading frame in *mdx* mice has also been observed in the heart.^184^ However, other studies show that unless sufficiently high levels of correction are achieved, CRISPR-induced dystrophin expression can be lost from skeletal muscles.^185^ This is partly due to the inability of conventional AAV vectors, such as AAV8 and AAV9, to target muscle satellite stem cells.^186^ While targeting satellite cells is disputable, they could potentially be modified *ex vivo*. Accordingly, Hicks et al.^187^ demonstrated that human iPSC-derived skeletal muscle progenitor cells, corrected with CRISPR/Cas9, restored dystrophin expression when transplanted into NGS-*mdx* mice muscles.^187^

More challenging for repair are duplications, which comprise 10%–15% of *DMD* mutations. Maino et al.^188^ generated mice with a long, 137 kb duplication spanning exons 18–30 of the *DMD* gene. When newborns of such mice were treated with a single sgRNA to induce excision of the duplication, full dystrophin expression was restored in up to 30% of cardiac fibers and 60% of skeletal muscle fibers.^188^ This study demonstrates that gene editing can be used not only in a way analogous to ASO-based exon skipping strategies, correcting small mutations, but also to ameliorate the effect of larger mutations in the *DMD* gene.

Importantly, the restoration of dystrophin expression has also been demonstrated in dystrophic dogs^189^ and pigs.^170^ AAV-mediated delivery of CRISPR/Cas9 restored dystrophin expression to up to 90% of the normal level in some skeletal muscles of DMD dogs, while in cardiac muscle, dystrophin level reached 92%.^189^ Moretti et al.^170^ showed the efficient restoration of truncated dystrophin in the hearts of DMD pigs, using AAV9 vectors to systemically deliver SpCas9 and sgRNAs to excise exon 51 (Table S4). In the majority of studies, the AAV vectors have been used for *in vivo* correction of DMD mutations. On the other hand, Gee et al.^190^ utilized nanovesicles to deliver Cas9 protein and sgRNA, demonstrating dystrophin gene editing in various human cells *in vitro*, including iPSC-derived neurons and myoblasts. This delivery tool was also tested in *mdx* mice to target the mutation in exon 23. Intramuscular injection induced exon 23 skipping, but the efficacy was low, not exceeding 1.5%. In contrast, Majeau et al.^191^ achieved a better outcome, with up to 20% deletion and excisions of exons 23 and 24, by delivering SpCas9 protein and sgRNA with extracellular vesicles.

BE and prime editing approaches have been applied to correct mutations in human iPSC-derived cardiomyocytes^192^^,^^193^ as well as in DMD animals.^193^^,^^194^^,^^195^ ABE and CBE performed single-swap editing by introducing single base pair changes at either the splice acceptor site (SAS) or the splice donor site (SDS) flanking a target region (Figure 6).^196^ This is possible because both ABE (which converts A-T base pairs to G-C) and CBE (changing C-G to A-T) can edit the canonical AG splice site of the SAS (which is CT on the antisense strand) or the canonical GT of the SDS, AC being on the opposite strand.^196^ Accordingly, CBE restored the reading frame by skipping exon 4 in DMD^E4∗^ mice, in which a 4 bp deletion was introduced in exon 4 by CRISPR/Cas9, editing the 5’splice site of exon 4.^197^ A significant increase in the life span of mice was noted, attributed to the amelioration of myocardial fibrosis and improvement of heart functions. The discovery of miniature nucleases, such as IscB proteins (with a size of about 400 aa), recently allowed the creation of a single AAV vector harboring the IscB.m16∗-CBE system. After intramuscular injection into DMD mice lacking exon 51, this system modified the splicing acceptor site (AG) adjacent to exon 50, causing its skipping and restoration of dystrophin expression in about 40% of fibers (Table S4).^198^ Of note, mini-dCas13X ABE, an RNA adenine base editor, has also been effectively used to correct the point mutation in another humanized mouse model of DMD.^199^

Prime editing has so far been tested only *in vitro*. It has been used for exon 52 reframing to restore the truncated dystrophin expression caused by the deletion of exon 51 in iPSC-derived cardiomyocytes.^193^^,^^200^ Modification of the splice donor site for exons 51 and 53 was used to correct two types of mutations in human myoblasts through exon skipping, connecting exon 50 to exon 53 and exon 44 to exon 54 by skipping exons 51 and 53, respectively.^201^

Overall, the efficacy of DMD gene editing often leads to higher dystrophin restoration than the level considered to exert a therapeutic effect, although the required level of dystrophin restoration sufficient to prevent muscular dystrophy remains debated. Some studies reported improvement with as little as 4% of normal dystrophin levels, while others suggest that reaching at least 15% may be necessary (reviewed by Godfrey et al.,^202^). The effectiveness tends to be lower when older animals with more extensive damage are treated, and the therapeutic effect may be more pronounced in the heart than in the skeletal muscles.^185^ Also, editing frequently leads to highly mosaic expression patterns, which likely impact overall efficacy.^203^ Whether this necessitates repeated gene editing (albeit less frequently than ASO therapy) or could be ameliorated by concomitant treatment with μDys gene therapy^203^ remains to be addressed in future studies.

### Delivery modes for gene editing

The originally used *Streptococcus pyogenes* (SpCas9) consists of 1,367 amino acids, and its cDNA is approximately 4,101 bp long. Therefore, together with regulatory elements and sgRNA sequences, it generally exceeds the 4.7 kb packaging capacity of AAVs. Accordingly, two AAV vectors are used, and a split SpCas9 is joined by the split-intein approach. The large size of SpCas9 was also the reason for earlier approaches that used adenoviral vectors rather than AAV.^204^ Moreover, the higher capacity of adenoviral vectors, namely gutless vectors, allows packaging and delivery of much bigger constructs, such as the BE or prime editing components.^205^ The advantage of the adenoviral vectors could be their short-term expression, while the drawbacks are high immunogenicity and lack of mature skeletal muscle or heart tropism, which can be achieved with different AAV vectors.

The introduction of Cas9 from *Staphylococcus aureus*, which consists of 1,055 amino acids (3,165 bp), enabled the use of a single AAV vector for SaCas9 expression and sgRNA delivery. However, SaCas9 is large enough compared with the AAV carrying capacity that modified versions of the enzyme, such as dCas9 combined with transcriptional activators, do not fit into a single AAV vector. The same is true for BE and prime editing systems. An even shorter Cas9 nuclease from *Campylobacter jejuni* (CjCas9), composed of 984 amino acid residues (2,950 bp), has also been utilized.^206^ Recent discoveries of miniature nucleases from other bacterial or archaeal species open possibilities for delivering these modified nucleases in one vector alongside an sgRNA. Furthermore, when two small nucleases with different PAM requirements are employed, relevant sgRNAs can be designed to target different genetic regions while the nucleases can be delivered in a single vector. Additionally, combining the nuclease-dead enzyme with active nucleases may allow targeting the same gene at two sites, for example, enhancing expression and excising inhibitory regions within that gene, respectively. We propose that this latter approach could potentially be applied to the upregulation of utrophin expression (discussed below). Finally, the use of short nucleases, with a cDNA length not exceeding 2,000 bp, may facilitate the construction of self-complementary AAV vectors, which could provide a quicker effect in transduced cells due to the omission of AAV double-strand DNA synthesis after transduction.^207^

Recent developments in non-viral approaches offer additional possibilities for delivering Cas mRNA or ready-to-use protein along with relevant sgRNAs using gold^208^ or lipid nanoparticles (LNPs).^209^ Wei et al.^209^ showed that intramuscular LNP injection containing Cas9 protein and an sgRNA resulted in the excision of exon 45, restoring the dystrophin reading frame in about 4% of fibers in ΔEx44 mice. Kenjo et al.^210^ demonstrated the feasibility of local intramuscular injection of LNPs targeting exon 45 in CRISPR/Cas9-generated ΔEx44 mice, which restored dystrophin expression in the injected hindlimb muscles. Systemic non-viral delivery has already been applied for the treatment of transthyretin amyloidosis,^211^ demonstrating its feasibility. Nevertheless, in the latter case, the target is the liver, while delivery to muscles and the heart may require enriching non-viral particles in proteins, allowing specific receptor-ligand interactions. The low immunogenicity of lipid nanoparticles, in contrast to AAV, may also allow repeated injection of the editing system.^210^ However, major improvements in targeting efficiency will be needed before adaptation of these non-viral systems to muscle disorders requiring bodywide gene delivery.

So far, the FDA has registered one CRISPR/Cas9-based gene therapy, which is Casgevy, the *ex vivo* treatment for sickle cell disease.^212^ Nevertheless, the *ex vivo* approaches would be rather ineffective in DMD until an efficient method of systemic delivery of myogenic cells is developed.

### Safety issues of gene editing

To date, clinical delivery of dCas9 has been attempted in only one DMD patient. The 27-year-old DMD non-ambulant man had a deletion of approximately 30 kb encompassing the muscle promoter and exon 1, resulting in the absence of the *Dp427m* isoform. However, the promoter and exon 1 of cortical (*Dp427c*) and Purkinje (*Dp427p1*) transcript variants were intact. The therapy was designed to upregulate the *Dp472c* expression using anAAV9 vector harboring dCas9 coupled to the VP64 transcriptional activator and was targeted with sgRNA to activate the *Dp427c* promoter (CRISPRa technology). Unfortunately, shortly after the intravenous injection of 1 × 10^14^ vg per kg body weight, the patient experienced a severe inflammatory response, leading to cardiac dysfunction, followed by acute respiratory distress syndrome and cardiac arrest 6 days post-treatment. The patient passed away 8 days after transgene delivery. The postmortem analysis did not detect antibodies against AAV9 or effector T cell reactivity, and the fatal outcome was attributed to a robust innate immune response against the AAV capsid.^213^

This tragedy points to the problems associated with gene therapy relying on the administration of high doses of AAV vectors. Preclinical studies in DMD animal models have demonstrated successful, if relatively inefficient, gene editing following systemic AAV delivery at doses ranging from 1–3 × 10^14^ vg/kg, which is the upper limit currently used in clinical studies. More efficient BE in the muscles was achieved with even higher doses, equivalent to 10^16^ vg/kg.^193^ However, such doses greatly exceed the upper limit of safety in humans. The immune response to high doses of the viral capsid poses a significant challenge in AAV-based gene therapy.^143^ Beyond this, there is also the risk of an immune reaction against the dystrophin protein, as observed in μDys gene therapy. A third concern potentially arises when intein-based AAV vectors are used to deliver editing constructs, as they may trigger an immune response against the intein. This is particularly relevant for BE or prime editing strategies, where the delivery of large constructs necessitates the use of split vectors. To mitigate these immune responses, muscle-specific promoters could be employed to restrict the expression of the gene editing complex to skeletal muscles and cardiomyocytes.^214^ Additionally, greater specificity for striated muscles could be achieved through the use of novel myotropic AAV serotypes.

Moreover, the crucial question is the time of gene editing. The majority of gene editing studies to date have been done using neonatal animals. Similarly, in the case of gene therapy with SMA, early intervention, before the onset of symptoms, has proven more effective. Likely, restoring dystrophin expression would also yield the best outcomes if performed before significant muscle damage, necessitating early diagnosis of DMD. However, this approach is not viable for older patients. Meanwhile, the studies performed in older dogs indicate that while editing is possible, its efficacy is markedly lower than μDys gene transfer.^203^ To compensate for this reduced efficacy, higher doses of gene editing vectors might be required, heightening the risk of exacerbated inflammatory and immune responses. Moreover, immune reactions against Cas9 were demonstrated to be stronger in older animals.^183^ Clearly, there is a great need for methods that can significantly improve the efficiency of gene editing in muscle, especially in mSCs.

These findings also suggest that applying editing approaches in younger patients is a rational strategy, provided that the associated safety issues are adequately addressed. The open question, which requires extensive preclinical research, is whether gene editing could be effectively combined with early diagnosis, enabling the initiation of therapy in presymptomatic patients. Animal studies suggest that gene editing in newborns can provide long-lasting effects, with benefits observed even several months after treatment (see examples in Table S4). However, if this approach proves effective, it should also be further adapted and made available for older patients to ensure broader therapeutic access.

Long-term persistence of gene editing is crucial if it is to be applied as a one-time treatment. Current studies suggest that sustained efficacy is more readily achieved in the heart than in skeletal muscle.^179^^,^^197^ This disparity is thought to be linked to contraction-induced injury and necrosis/regeneration of the latter and inflammation progressing faster than in the heart. Since cardiomyocytes are far more long-lived in DMD than skeletal myofibers, inefficient gene editing leads to a much more rapid loss of edited cells in skeletal compared with cardiac muscle cells.

A primary concern with gene editing is the risk of sequence- and tissue-specific off-target effects. Optimizing sgRNA design can reduce sequence-dependent effects, while utilizing muscle-specific vectors, and promoters can enhance tissue specificity. Lastly, a critical safety issue unique to gene editing is its irreversible nature. So far, this concern has not been addressed, but it will become increasingly relevant as *in vivo* gene editing moves closer to clinical application.

### CRISPR/Cas for modulatory therapy

Liao et al.^215^ used the CRISPR approach to epigenetically modulate the expression of genes that could potentially improve the phenotype of *mdx* mice. They demonstrated that upregulation of *klotho* expression, a gene known to be epigenetically silenced in the muscle of *mdx* mice, as well as the induction of utrophin expression, ameliorated the DMD phenotype.

It has also been found that natural exon skipping, caused by maternally inherited *trans*-generational epigenetic silencing of the Celf2a splicing factor, results in partial restoration of dystrophin expression.^216^ Investigation of the iPSC-derived myoblasts, in which Celf2a was inactivated by CRISPR/Cas9, leading to the amelioration of DMD, offers a new treatment approach for this disease.^216^ Finally, the CRISPR/Cas9 method seems particularly promising for the upregulation of utrophin expression.

## Genetic strategies for upregulation of utrophin expression

Utrophin is a paralog of dystrophin, encoded by the *UTRN* gene located on chromosome 6 in humans and on chromosome 10 in mice.^217^^,^^218^ The size of its gene and the cDNA are similar to dystrophin, and they share a very similar exon-intron organization. Like dystrophin, utrophin is expressed in several isoforms, with two main, utrophin A and utrophin B, 395 kDa proteins. As with dystrophin, there are also shorter isoforms, namely Up140, Up113 (G-utrophin), and Up71. Utrophin shares 80% sequence identity with dystrophin, with homologous domains responsible for the cytoskeleton-ECM connection.^219^^,^^220^ The protein is primarily synthesized in skeletal muscles during the fetal period, and its presence in a healthy adult organism is restricted to the neuromuscular junction (NMJ) and the myotendinous junction (MTJ).^221^^,^^222^ Utrophin A is expressed in skeletal muscles, while utrophin B is expressed in endothelial cells, and they are regulated by different, mostly cell-specific promoters.^223^^,^^224^^,^^225^ Utrophin forms a utrophin-glycoprotein complex (UGC), analogous to dystrophin (DGC).^226^ Despite the limited expression in adult muscle, the important role of utrophin is exemplified by the properties of double knockout mice (*mdx/utrn*^−/−^), lacking both dystrophin and utrophin, which are significantly affected and demonstrate a severe dystrophic phenotype.^227^^,^^228^ On the other hand, the mild phenotype of *mdx* mice is thought to be linked to postnatal utrophin upregulation, which does not occur frequently in humans. A high level of utrophin in *mdx* mice can compensate for the lack of dystrophin,^229^^,^^230^ while in humans, it does not reach the levels required to prevent the disease progression.^217^^,^^222^ Accordingly, some suggest that the severity of DMD phenotype in humans might inversely correlate with utrophin upregulation^231^^,^^232^^,^^233^; however, a lack of such association has also been demonstrated.^234^

**Figure 7:**
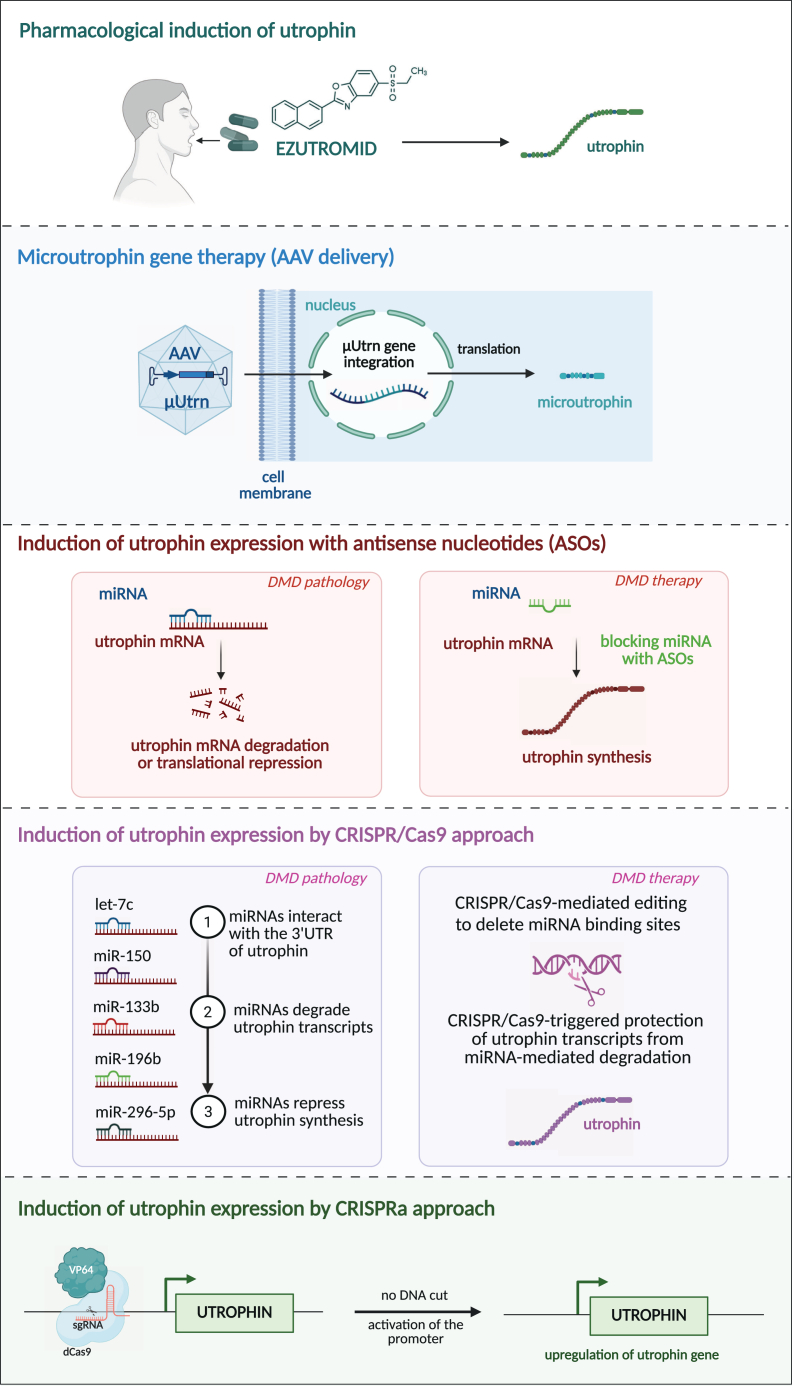
Utrophin-based therapy for DMD Approaches to stimulate utrophin expression include small molecules (e.g., ezutromid), gene therapy strategies aimed at restoring utrophin or overexpressing micro-utrophin (μUtrn) in muscle tissue, and gene editing approaches using antisense oligonucleotides (ASOs), CRISPR/Cas9, or CRISPRa technologies.

Based on these factors, restoration of utrophin expression has been proposed as a promising therapeutic modality for DMD. The rationale for this strategy relies on the following facts: (1) utrophin is a paralog of dystrophin; (2) it is capable of replacing dystrophin as an anchor of sarcolemma to the cortex cytoskeleton; (3) it is non-immunogenic when overexpressed, in contrast to dystrophin, which may be recognized as a foreign protein by the immune system of DMD patients; and (4) it is potentially useful regardless of a patient’s *DMD* mutation. Various methods for utrophin overexpression have been tested, including stimulation with small molecules, epigenetic modifications, post-transcriptional regulation, and gene editing (Figure 7) (reviewed by Roberts et al.^235^). Among the pharmacological agents tested, ezutromid, an orally bioavailable compound, yielded promising pre-clinical results in animal models.^236^ However, it failed to meet its primary and secondary endpoints in DMD patients.^237^^,^^238^ It is worth mentioning that the replacement of dystrophin by utrophin has several limitations, as utrophin does not have the same functional capacities as dystrophin. Despite sharing significant sequence similarity with dystrophin, it is unable to anchor nNOS to the sarcolemma,^239^ a crucial function of dystrophin that may impact its therapeutic properties. Additionally, the binding of utrophin to β-DG is weaker than dystrophin,^240^ which may further limit its effectiveness in stabilizing the muscle membrane.

The rationale supporting the therapeutic modality of utrophin originated from studies in transgenic dystrophic mice, overexpressing truncated utrophin, which showed improvements in muscle function.^241^^,^^242^ Further progress has been made with the expression of full-length utrophin, which also led to functional enhancements.^243^ Similarly to dystrophin, short forms of utrophin are the obvious candidates for gene therapy in DMD patients due to the capacity of AAV vectors. Our research (Chamberlain’s team) demonstrated that intravenous delivery of AAV6 vectors expressing microutrophin (μUtrn) increased lifespan and improved muscle function in *mdx/utrn*^−/−^ mice.^244^ Alleviation of cardiac and skeletal muscle defects has also been demonstrated by AAV9-μUtrn gene therapy in D2/*mdx* mice.^245^ In accordance with the presence of utrophin during fetal development, the μUtrn gene therapy ameliorated muscular dystrophy in both *mdx* mice and golden retriever muscular dystrophy (GRMD) dogs, without inducing immunity, in contrast to the strong T cell response observed when μDys was expressed from a non-muscle (ubiquitous, CMV) promoter.^246^ Interestingly, in DMD patients, who still express some utrophin, the potential unfavorable interaction between full-length utrophin and short, exogenously introduced forms must be considered. Accordingly, μUtrn gene therapy has been demonstrated to offer better protection in dystrophin/utrophin double knockout mice than in *mdx*^4cv^ mice, which still express some utrophin.^247^ Furthermore, recent studies on intein-mediated restoration of full dystrophin^158^^,^^161^ present the possibility of applying a similar approach to utrophin gene therapy.

CRISPR/Cas9 upregulation of utrophin expression has been demonstrated in *mdx* mice, in which the shortened sgRNAs, unable to activate nuclease Cas9 activity, upregulated the *Utrn* promoter.^215^ In a similar approach, catalytically inactive Cas9 upregulated utrophin expression in myoblasts derived from DMD patients.^248^ Recently, our team (Dulak’s) established an effective strategy to activate the *UTRN* promoter in human induced pluripotent stem cell (hiPSC)-derived cardiomyocytes, using catalytically inactive Cas9 linked to the VP64 transactivation domain (dCas9-VP64).^249^ The therapeutic potential of utrophin has also been exemplified by a study in which DMD iPSCs were corrected by *Sleeping Beauty* (SB) transposon-mediated overexpression of utrophin.^250^ The SB non-viral integrating system combines the advantages of viral vectors and naked DNA, providing long-term gene expression while maintaining safety features.^251^ Transplantation of skeletal muscle progenitors, differentiated from SB-modified DMD iPSCs, into the muscles of dystrophic mice resulted in efficient cell engraftment, improved contractile strength, and restoration of muscle fiber repair machinery.^250^ The expression of *UTRN* is subjected to extensive regulation at both transcriptional and post-transcriptional levels. Targeting the utrophin promoter was the first modality to be verified. In addition to small molecules, heregulin, the protein recognized to activate the GA-binding protein (GABPa/b) transcription factor complex in neuromuscular junctions, has also been tested, demonstrating improvement of the mechanical properties of muscles of *mdx* mice.^252^

On the other hand, the utrophin promoter activity is inhibited extrasynaptically at various levels, including the Ets-2 repressor.^253^ Accordingly, the knockdown of this repressor by small interfering RNA enhanced utrophin mRNA in muscle cells *in vitro*.^253^ Posttranscriptional silencing of utrophin is mediated by the microRNAs targeting the 3′UTR region, which hosts multiple repressive micro RNA (miRNA) binding sites (such as let-7c, miR-150, miR-196b, miR-296-5p, and miR-133b).^254^^,^^255^ Thus, inhibiting the miRNA effect either by let-7 targeting oligonucleotides^254^^,^^256^ or by CRISPR/Cas9 editing of five miRNA binding sites^255^ upregulated utrophin expression in *mdx* mice muscles. Whether the same approaches will work in human cells remains to be established.

## Treatment of DMD cardiomyopathy

DMD is primarily associated with impairments in skeletal muscle function, but cardiomyopathy is the leading cause of premature death in DMD patients, typically occurring in their 30s. Currently, there is no curative treatment for DMD-related heart failure, highlighting the critical need to identify novel therapeutic avenues and gain deeper mechanistic insights (reviewed by Florczyk-Soluch et al.^257^). Moreover, heart dysfunction also affects some carrier females.^258^^,^^259^^,^^260^^,^^261^ In addition, the rare condition known as X-linked dilated cardiomyopathy (XLDCM) can manifest in males who harbor certain mutations in the *DMD* gene, and they display low dystrophin expression in skeletal, but not cardiac, muscles, resulting in a severe cardiomyopathy without overt skeletal myopathy.^262^ Therefore, understanding the molecular mechanisms underlying cardiac dysfunction due to DMD mutations is of utmost importance for the development of effective therapies for dystrophin-dependent cardiomyopathies. Investigating DMD cardiomyopathy and potential therapies is particularly challenging due to the historical insufficiency of suitable animal models, a challenge that is even more pronounced than in the case of skeletal muscle research. In *mdx* mice, heart dysfunction typically develops only very late in life and does not reach the same severity seen in DMD patients.^263^ Additionally, dilated cardiomyopathy has been observed only in aged female *mdx* mice^264^ and female *mdx*^4cv^.^265^ Nonetheless, the recently described Fiona DMD mouse model discussed earlier provides a new model for DMD-associated heart failure in mice. This relative lack of good animal models represents a limitation, as female DMD patients are extremely rare, making it difficult to fully replicate the disease’s progression and understand the associated cardiac pathology in the context of sex differences.

The absence of dystrophin in cardiomyocytes leads to similar pathological changes as those observed in skeletal muscle cells, including dysregulation of Ca^2+^ balance, an increase in ROS levels, and impaired mitochondrial function.^54^ In the hearts of individuals with DMD, the deficiency of dystrophin results in compromised contractile function of cardiomyocytes. However, it is important to note that dystrophin has also been detected in vascular smooth muscle cells, endothelial cells, and fibroblasts,^55^^,^^56^^,^^57^^,^^58^ although the level and significance of its expression in these latter two cell types raise concerns. Diagnosing heart dysfunction in DMD patients can be challenging, as some individuals do not exhibit obvious signs of heart failure, especially since many use wheelchairs and do not experience an elevated cardiac workload. Unfortunately, as of now, there is no effective treatment for dystrophin-dependent cardiomyopathy, which is ultimately fatal for DMD patients and also poses significant risks for BMD individuals and some female carriers.^266^ Theoretically, the same therapeutic approaches developed for skeletal muscle could also apply to addressing impaired heart function in DMD patients. However, treating the heart carries additional challenges, as the effectiveness of various delivery modalities differs between skeletal muscles and the heart. Concerning genetic therapies, it has been demonstrated that the efficacy of ASOs in the murine skeletal muscles is not directly comparable to their effects in the heart, as discussed above (see Duan’s review for more details^267^). However, this has not yet been assessed in humans. A similar concern may apply with AAV gene therapies, as the delivery efficiencies to the heart and skeletal muscles might differ. When AAV containing the μDys gene is delivered systemically, improvements in both skeletal and cardiac function are expected. This has indeed been observed in young *mdx* mice^68^^,^^124^^,^^268^^,^^269^ and even in older mice aged 16–20 months.^270^ However, this approach might be problematic in the advanced stages of disease in DMD patients. Indeed, in 21-month-old *mdx* mice, AAV-mediated μDys gene therapy alleviated stress-induced cardiac death but did not significantly reduce myocardial fibrosis.^271^ Additionally, when only skeletal muscle function was rescued with μDys in *mdx* transgenic mice, no significant effect on cardiomyopathy was observed, neither alleviating nor exacerbating the condition.^272^ Other studies in both mice and patients have shown a clear aggravation of cardiomyopathy when dystrophin is produced solely in skeletal muscles.^93^^,^^273^ This underscores the critical need to treat both skeletal and cardiac muscles in DMD patients to effectively manage the disease’s progression and its cardiac complications.

A recent study by Hart et al.^274^ raised some concerns about potential deleterious effects of excessively high μDys expression in the heart. Treatment of 1-month-old D2/*mdx* mice, which develop cardiomyopathy at 12 months of age, with four different AAV-μDys constructs (some of which are used in clinical trials), resulted in high expression levels in skeletal muscles. Notably, there was a 10-fold higher expression in the heart compared to the skeletal muscles. Although therapy resulted in partial improvement of the skeletal muscle function, it also led, in some cases, to the lethal acceleration of cardiac disease at 12 and 18 months of age, with two out of the four μDys investigated. However, such a negative effect was not observed in the other two μDys. One of the non-toxic vectors was used in the Pfizer clinical trial, and the other in the Solid Biosciences trial, with the latter carrying the nNOS-localization domain.^125^ The authors suggest that the observed detrimental effect was due to the competition between μDys and utrophin expressed in cardiomyocytes, resulting in utrophin displacement, although no adverse events have been noted in other studies with μDys expressed in *mdx* hearts or in dystrophin/utrophin double knockout hearts.^132^^,^^133^^,^^244^ Hart et al.^274^ warn about the potential risk of such toxic effects in patients, although it has to be noted that in their study, they used high doses of AAV vectors, and the study was performed in D2.*mdx* mice, which may reveal additional heart damage due to calcification present already in original wild-type strain.^275^ However, another study showed that cardiac toxicity occurred only when miniDys was overexpressed at extremely high levels—about 100 times the normal amount. In contrast, when full-length dystrophin was expressed at levels 50 times higher than normal, it was not toxic, despite these hearts were also expressing utrophin.^276^^,^^277^ Accordingly, the long-term effect of μDys gene therapy on the heart of DMD patients has to be considered, especially as any adverse effects may not become apparent until years after gene delivery, when cardiomyopathy typically begins to develop as the disease progresses. Importantly, in a recent study, Sherlock et al.^278^ reported no significant cardiac adverse events from miniDys gene therapy in ambulatory DMD participants. The analysis, based on a phase 1b, open-label trial (NCT03362502), monitored cardiac biomarkers and parameters, including cTn-I levels, ECGs, cardiac MRI, CK-MB fraction, pulse rate, and blood pressure, over the year following administration of fordadistrogene movaparvovec. The findings demonstrated no prominent cardiac complications associated with the therapy. However, longer-term follow-up is necessary to fully assess the risk of potential delayed cardiac adverse events. Supporting this need, Forand et al.^279^ reported that while μDys significantly improved survival and cardiac function after one year of treatment in *mdx/utrn*^−/−^ mice, the therapy resulted in signs of cardiac inflammation and increased septal thickness.

Moreover, improvements in skeletal muscle function and increased physical activity may inadvertently increase the load on the heart, potentially accelerating cardiomyopathy. However, the data on the expression of μDys in the hearts of patients involved in clinical trials is currently unknown (for further references see:^274^). Many other studies have not reported cardiac toxicity from high levels of μDys expression in hearts,^68^^,^^125^^,^^280^ including patients in clinical trial.^277^ In addition, beneficial cardioprotective effects of AAV-μUtrn gene therapy have been demonstrated in *mdx* mice, both in models of acute injury and maladaptive cardiac remodeling in response to pharmacologic and exercise-induced cardiac stress tests.^281^ Further studies will be essential to clarify the potential risks and therapeutic advantages of these approaches in human clinical trials.

An efficient approach for the treatment of cardiomyopathy in the dystrophic pigs, obtained by deleting exon 52,^282^ has been proposed by Moretti et al.^170^ Restoration of shortened dystrophin expression, devoid of exons 51 and 52, was achieved by AAV9 delivery of Cas9 and relevant sgRNAs, enabling excision of exon 51. This led to widespread dystrophin expression across limb muscles, the diaphragm, and the heart. The survival of these pigs (the majority of which, when untreated, died within the first week after birth, with none surviving beyond 105 days) was significantly increased up to 136 days.^170^ In a subsequent study, another pig model that mimics BMD was created by deleting exon 51 in *DMD*Δ52 pigs (as skipping DMD exon 51 in subjects lacking exon 52 can restore dystrophin expression). In comparison to animals lacking exon 52, which exhibited significantly reduced cardiac function at 3.5 months of age, the additional deletion of exon 51 completely rescued the cardiac deterioration.^283^

## Genetic therapies and modulatory genes—a few potential targets

### Calcium and iron handling

Calcium handling is significantly disturbed in DMD.^284^ Because impaired calcium handling might be linked to reduced activity of sarco/endoplasmic reticulum calcium ATPase (SERCA2) (the calcium pump that transports calcium from cytosol to the sarcoplasmic reticulum), studies have examined its overexpression in mice. AAV9-SERCA2 treatment of 3-month-old *mdx* mice resulted in the amelioration of the cardiomyopathy and overall significant improvement up to 18 months. Treated animals showed enhanced motor activity as measured by the grip force test and treadmill performance, myocardial fibrosis was prevented, and cardiac electrophysiological activity was normalized.^285^ Additionally, a recent study by the Duan group showed improvement of muscle function in dystrophic dogs after AAV-mediated SERCA2 therapy.^286^ In this approach, enhanced sarcoplasmic reticulum calcium uptake and better various physiological parameters in dystrophic dogs were evident without improvement in muscle histology. Similar effects have also been observed by AAV gene therapy with DWORF, a positive regulator of SERCA function,^287^ which is also reduced in *mdx* mice. Accordingly, AAV9 overexpression of this regulator ameliorated DMD cardiomyopathy in dystrophic mice injected at 6 weeks of age, and the effect persisted up to 18 months of age.

Interestingly, improved DMD pathogenesis could potentially be achieved by inhibiting the activity of specific genes that are upregulated due to the disturbances caused by the absence of dystrophin and utrophin. Recently, Mareedu et al.^288^ demonstrated that reducing the expression of sarcolipin (SLN), a potent inhibitor of SERCA pump, by heterozygous knockout, significantly improved mitochondrial structure and the interaction between mitochondria and the sarcoplasmic reticulum in cardiomyocytes of dystrophin-utrophin double knockout mice. Similarly, AAV9-mediated RNA interference of SLN expression attenuated muscle pathology and improved skeletal muscle, diaphragm, and cardiac function in *mdx*/*utrn*^−/−^ mice.^289^

Interestingly, these effects also paralleled changes in metabolism, including improved glucose tolerance and enhanced insulin sensitivity, as observed in *mdx* mice lacking either one or two alleles of the *SLN* gene.^290^ Recently, our (Dulak’s) study revealed impaired iron handling in human DMD iPSC-derived cardiomyocytes.^164^ These DMD cardiomyocytes demonstrate a lower level of mitoNEET, the mitochondrial outer membrane protein that removes iron from mitochondria to the cytoplasm. The other genes of the iron metabolic pathway were also affected. Restoration of dystrophin expression by CRISPR/Cas9 gene editing normalized mitoNEET level in DMD cardiomyocytes and improved iron handling. Further studies are necessary to assess whether this pathway can be the target of modulatory therapy.

Of note, our (Dulak’s) other studies showed that heme oxygenase-1 (HO-1), the inducible heme-degrading enzyme, is upregulated in the muscles of *mdx* mice,^44^ which could potentially be linked to increased iron release. On the other hand, the expression of HO-1 (encoded by the *Hmox1* gene) is decreased in dystrophic mSCs.^44^ We have previously demonstrated that a lack of HO-1 accelerates the differentiation of mSCs due to the higher expression of myomiRs.^291^ Thus, one can presume that the lower expression of HO-1 in DMD satellite cells might be associated with their accelerated and disturbed differentiation.^44^ Accordingly, we recently showed that transgenic *mdx* mice overexpressing *HMOX1* in mSCs exhibited improved properties and prevented muscle damage.^292^

### Vascularization

Blood vessel formation is known to be affected in DMD (reviewed by Podkalicka et al.,^293^). Angiogenesis has been demonstrated to be impaired in *mdx* muscles, the effect ascribed to the lack of expression of dystrophin in endothelial cells, where it may form a complex with eNOS and caveolin-1.^56^ Indeed, a decrease in eNOS expression in arterial endothelial cells lacking dystrophin has been described by Kodippili et al.^294^ in DMD dogs. However, the expression of dystrophin in endothelial cells may be very low (discussed above and our unpublished data). Nevertheless, it appears that impaired angiogenic properties could also affect myoblast differentiation.^56^ Interestingly, as we have also demonstrated, the compromised revascularization of the *mdx* muscle may be age-dependent.^295^

Vascular endothelial growth factor (VEGF), a key angiogenic growth factor, has also been implicated in myogenesis.^296^ VEGF has been overexpressed from AAV vectors by direct intramuscular administration in *mdx* mice, leading to enhanced muscle regeneration, linked with an increased number of mSCs, decreased muscle fibrosis, and, consequently, improved forelimb strength. Additionally, the proangiogenic effect was observed in regenerating muscles.^297^ Also, intravenous delivery of AAV9-VEGF in the *mdx* mice demonstrated an improvement of muscle damage and increased muscle force.^298^ The proangiogenic effect has also been achieved by Sonic hedgehog (SHH) gene therapy in *mdx* mice,^299^ which led to increased insulin-like growth factor 1 (IGF-1) and VEGF expression. Both factors contributed to better muscle regeneration, enhanced vascularization, and decreased fibrosis.

Angiogenesis can be affected not only by decreased levels of VEGF but also by impairment of its activity. Higher levels of soluble VEGF receptor-2 (encoded by the *Flt1* gene) can attenuate the proangiogenic effect of VEGF. Accordingly, Verma et al.^300^ demonstrated that while global knockout of the *Flt1* gene is detrimental for *mdx*/*utrn*-deficient mice, endothelial-specific removal of the *Flt1* receptor increased vascular density and the number of satellite cells and consequently improved the *mdx* skeletal muscle phenotype. This effect was recapitulated by the treatment of *mdx* mice with Flt1-blocking peptides or monoclonal antibodies.^301^ However, not all proangiogenic effects are beneficial. In another study, Asakura’s group showed that inhibition of miR-92a increased blood vessels and mSCs in skeletal muscles of *mdx* mice; however, muscle regeneration was not improved, probably because miR-92a is essential for proper mSC differentiation.^302^

### miRNAs as the target for DMD therapy

Non-coding RNAs, such as miRNAs and lncRNAs, play crucial roles in regulating gene expression, and their dysregulation has been increasingly implicated in DMD (for a review, see:^303^). Due to their multiple targets, the effect of a particular miRNA can vary and could be cell- and tissue-dependent. In DMD, changes in miRNAs associated with inflammation, fibrosis, mSC function, and differentiation have been found. Notably, miR-146a, a key inflammation-related miRNA, is increased in DMD muscles, and treatment with corticosteroids decreases the inflammatory miRNA transcriptome.^110^ However, our findings indicate that the absence of miR-146a in *mdx* mice does not significantly aggravate disease conditions, indicating that its increased expression in DMD might not be protective.^52^ Moreover, miR-146a appears to target dystrophin, as a binding site for this miRNA is present in the 3′UTR of dystrophin mRNA, similar to some other miRNAs, like miR-374a and miR-31.^109^ This can lead to decreased expression of truncated dystrophin present in BMD patients, potentially aggravating the disease. Moreover, when the shortened dystrophin expression is restored by exon skipping, the increased level of dystrophin-targeting miRNAs may abolish this therapeutic effect. Accordingly, it has been recently demonstrated that deletion of miR-146a enhances therapeutic dystrophin restoration in *mdx*52 mice, in which the skipping of exon 51 restores the reading frame.^111^

Our (Dulak’s team) studies revealed that miR-378a, an abundant miRNA in the heart and skeletal muscles that also regulates metabolism (for references see review by Krist et al.^304^), paradoxically plays a detrimental role in dystrophic mice. *Mdx* mice lacking miR-378a demonstrated better exercise capacity and improved skeletal muscle inflammation than animals deficient in dystrophin only.^305^ Interestingly, deleting miR-378a improved overall metabolism, as *mdx*/miR-378a^−/−^ mice displayed better glucose tolerance and insulin sensitivity, normalized liver glycogen levels, and increased systemic metabolism compared to *mdx* mice.^306^ MicroRNAs are also essential for controlling stem cell activity and muscle regeneration. Recently, Fukuoka et al.^307^ showed that systemic administration of miR-199-3p mimics to *mdx* mice markedly improved their muscle strength. Interestingly, miR-199 has also been demonstrated to stimulate cardiomyocyte proliferation, and AAV-mediated delivery of miR-199a ameliorated myocardial infarction in mice^308^ and pigs.^309^ The suppression of fibrosis and restoration of muscle function have been observed in dystrophic animals treated concomitantly with AAV vectors containing μDys and miR-29c.^310^ As mentioned above, inhibition of miR-92 improved vascularization and the number of mSCs in *mdx* mice, although the lack of an effect on muscle regeneration indicates the complexity of microRNA activity, due to their multiple targets.^302^ It remains to be investigated whether genetic approaches or pharmacological treatments affecting the miRNA levels could be elaborated for the modulatory therapy of DMD.

### Other genes as the targets for DMD therapy

Recent studies reveal additional potential pathways to modulate DMD severity and improve the patient’s quality of life. Paradoxically, some skeletal muscles, like extraocular ones, are less affected by dystrophin deficiency. Novel studies reveal various genes responsible for the observed differences. Accordingly, Taglietti et al.^311^ have shown that thyroid-stimulating hormone receptor (TSHR) is increased in the muscles of DMD rats, which prevents muscle senescence. The effect was mimicked by forskolin treatment, an adenylyl cyclase activator, which stimulated TSHR signaling, enhanced mSC proliferation, and reduced their senescence, leading to improved overall performance of DMD rats.^311^ It remains to be investigated whether this or e.g., Fhl2 LIM-protein, which rescued the dystrophic phenotype in the zebrafish DMD *sapje* model,^312^ could be elaborated for therapeutic purposes in DMD patients.

Another modulatory gene being tested in the clinic is *GALGT2*, the cytotoxic T cell N-acetylgalactosamine transferase. It has been shown to protect against the loss of skeletal muscle function^313^ and preserve heart function in aging *mdx* mice. The cardioprotective effect was correlated with increased glycosylation of α-dystroglycan and elevated utrophin levels.^314^ A similar effect, although without improvement in muscle function, has been observed in GRMD dogs.^315^ The phase 1/2 open-label dose escalation clinical study with rAAVrh74 expressing GALGT2 was initiated at Nationwide Children’s Hospital in Columbus, Ohio.^316^ Only two patients (6.9 and 8.9 years of age) were enrolled and injected with 5.0 × 10^13^ and 2.5 × 10^13^ vg rAAVrh74.MCK.GALGT2/kg per leg. Despite pre-existing antibodies to the rAAV capsid and modest T cell activity against rAAV and the GALGT2 protein, no significant liver damage was identified. A differential response to the treatment was observed as patient 1, treated earlier and at a higher dose, showed improvement with better 6MWT, 100 m run time, and NSAA scores, whereas patient 2 exhibited a decline. More studies are needed to draw meaningful conclusions and confirm the observation that the younger patients experience better improvement with a higher dose.^316^

## Why are genetic therapies for DMD less effective than for SMA?

Genetic therapies based on ASOs and AAV-gene delivery have been demonstrated to be successful in patients with spinal muscular atrophy (SMA), the disease caused by the mutation of the survival motor neuron 1 (*SMN1*) gene. While these therapies are not curative, they can provide significant benefits for many patients. Notably, Zolgensma, an AAV-based gene therapy delivering the *SMN1* gene, has saved the lives of many SMA type 1 patients and has significantly improved their quality of life.^317^^,^^318^ However, the situation is so far different in the case of DMD. Although ASOs and, more recently, gene therapy for DMD have been approved by the FDA, their real effectiveness remains unproven and is a subject of ongoing debate.

Despite the extensive understanding of SMA and DMD, these diseases and, hence, the potential effectiveness of therapies are different. First, dystrophin is a big protein, encoded by the largest known human gene, while SMN is much smaller. Accordingly, the gene therapy aimed at restoring the full protein is much more challenging and currently nearly impossible for DMD, while for SMA, AAV vectors can be effectively used to deliver the complete coding sequence. Second, DMD is caused by hundreds of different mutations in the DMD gene, and some available treatments, such as ASOs, are so far mutation-specific, making them unsuitable for all patients. In SMA, this issue does not exist. The full SMN protein can be expressed by either AAV-SMN1 delivery or exon-inclusion ASO targeting SMN2, allowing these therapies to be accessible to all affected individuals. While AAV-μDys gene therapy can be applied to all patients who do not have antibodies against AAV, the overall effectiveness and applicability of therapies involving small and not fully functional dystrophin continue to be a challenge. Furthermore, AAV-μDys trials have consistently shown highly mosaic patterns of dystrophin expression, and DMD may require higher and more uniform expression than what conventional AAV vectors and clinically safe doses can achieve. Another factor to consider is the immune response to gene therapy. In many cases of DMD, the dystrophin protein is not expressed at all, while, in SMA patients, the SMN protein is produced, albeit in small amounts, from the dysfunctional *SMN2* gene. Accordingly, there is no concern about an immune response against the therapeutic protein in SMA, unlike in some DMD individuals. Finally, DMD symptoms typically do not overtly manifest soon after birth, whereas early onset is common in SMA patients. As an effective treatment for SMA is available, postnatal diagnostic screening can identify affected newborns, enabling the therapy to be initiated even in asymptomatic patients. This early intervention significantly improves the chances of a positive therapeutic outcome. In contrast, current therapies for DMD have been applied to patients with already developed or advanced diseases. It remains unclear whether initiating therapy in DMD boys before the onset of muscle pathology and without clinical signs would be effective.

## Conclusions and perspectives

The development of effective therapies for DMD has not yet been achieved. However, advancements in gene therapy offer hope, particularly with the development of improved gene therapy vectors that can efficiently deliver dystrophin to skeletal, respiratory, and cardiac muscles. Also, targeting other affected organs, in particular the nervous system, as DMD affects the neurological development of at least one-third of DMD boys, should not be overlooked. It remains to be confirmed whether restoration of shortened dystrophin by exon skipping or AAV-based μDys gene therapy is the more effective approach to improving the health conditions of the patients, and both methods would benefit greatly by being able to produce higher dystrophin levels.

Novel, intein-based strategies to deliver full-length dystrophin, especially in conjunction with myotropic AAVs, hold promise as a more efficient approach. A key consideration for these gene therapies is the timing of their administration. It will be important to establish whether these genetic therapies can be initiated early in life, such as before symptoms appear, and whether and how often they need to be repeated, especially if dystrophin expression diminishes over time. Starting genetic therapies early would require newborn screening, which raises ethical concerns when effective or partially preventive therapies are not yet available. Moreover, the use of AAV gene therapy poses the risk that its effect may decrease as the patient grows, since transgene expression might be lost with increasing muscle weight, and repeated AAV injections will become problematic due to potential immune responses to the vector. The bigger the patient, the higher the vector loads will be necessary, which will also increase the risk of adverse events. On the other hand, even if the beneficial effects will disappear with time, early application of effective gene therapy may stabilize the condition and delay health deterioration.

The development of effective genetic approaches that could be safely and repeatedly administered to patients remains a rational approach to further develop. It is also likely that a significant improvement in patient survival and quality of life will require more effective combinatorial therapies, involving both the restoration of dystrophin and/or the upregulation of utrophin, coupled with modulatory therapies addressing secondary pathological features.

An important issue is also the availability of therapies for all patients. The current costs of ASOs are very high, and the expenses associated with gene therapy are even more substantial. Long-term continuation of these therapies will create a burden for the insurance systems. It is worth noting that none of the available ASOs are registered in Europe, primarily due to concerns about whether they work. Nevertheless, we are at a time of better understanding of disease mechanisms and improvements in effective genetic approaches for restoring dystrophin expression. If these strategies prove to significantly ameliorate muscle and heart damage, then early diagnosis and the initiation of treatment may offer greater hope for long-term successful outcomes.

## Acknowledgments

Supported by the grants from the National Science Center: MAESTRO #2018/30/A/NZ3/00412 and DAINA #2024/52/L/NZ3/00142 to J.D. and OPUS #2019/35/B/NZ3/02817 to A.Ł. J.D. was a 2024 STEM Impact Award Fulbright fellow at the University of Washington Senator Paul D. Wellstone Muscular Dystrophy Specialized Research Center. J.S.C. was supported by 10.13039/100000002NIH grant R01 AR40864-35 and a grant from the 10.13039/100005202Muscular Dystrophy Association (USA). Figures were created with BioRender.com.

## Author contributions

A.Ł. and J.D., conceptualization; A.Ł., J.S.C., and J.D., writing and editing; A.Ł., figure conceptualization and drawing; A.Ł., J.S.C., and J.D., funding acquisition.

## Declaration of interests

A.Ł. and J.D. declare that they have no conflict of interest and have no relationships with the industry. J.S.C. is an inventor and holds intellectual property related to numerous micro- and mini-dystrophins and CK-based regulatory cassettes, and he also holds equity in and is a member of the scientific advisory board for Solid Biosciences and KineaBio.

## Supplemental information

Document S1. Tables S1–S4

Document S2. Article plus supplemental information
