# Supplementary material for: Genetic strategies for therapy of Duchenne muscular dystrophy
Source: Mol Ther Nucleic Acids. 2025 Oct 31;36(4):102759. doi: 10.1016/j.omtn.2025.102759 (PMC12664987; doi:10.1016/j.omtn.2025.102759)
Supplement: Document S1. Tables S1–S4 [file mmc1.pdf]

OMTN, Volume *36*

Supplemental information

Genetic strategies for therapy of Duchenne muscular dystrophy

Agnieszka Łoboda, Jeffrey S. Chamberlain, and Józef Dulak

Table S1. Comparison of Duchenne and Becker muscular dystrophy.

	**DMD**<br>**Duchenne muscular dystrophy**	**BMD**<br>**Becker muscular dystrophy**
**Etiology**	X-linked recessive disease, caused by mutations in the *DMD* gene	
**Dystrophin protein**	absent or extremely low level,<br>non-functional	shortened protein and reduced levels,<br>but protein is functional
**Type of mutation***	out-of-frame<br>60-70% deletions;<br>5-15% duplications;<br>20% point mutations, small deletions, insertions	in-frame<br>60-70% deletions;<br>20% duplications;<br>5-10% point mutations, small deletions, insertions
**Incidence**<br>**(among male births)**	1 in 5,000	1:16,700-1:18,500
**Age of onset**	early onset, 2-5 years	late-onset ~ 10-12 years
**Disease progression**	fast	slow
**Ambulation**	approximately till 9-11 years	beyond 15-20 years
**Life expectancy**	~30 years	~ 40-60 years
**Diagnostics**	creatine kinase, genetic tests to confirm *DMD* mutation	
**Treatment**	mostly supportive	
**Central nervous system**	quite common cognitive dysfunction,<br>with deficits in verbal, short-term, and working memory	rare incidence of central nervous system abnormalities
**Respiratory failure**	common; progressive increased respiratory load<br>and respiratory muscle weakness	uncommon; respiratory functions and spirometry<br>are almost within normal values
**Metabolic alterations**	quite common, including obesity,<br>insulin resistance, glucose intolerance	less common than in DMD patients
**Cardiomyopathy**	very common; the leading cause of death;<br>it generally follows the skeletal muscle defects	might be evidenced before<br>skeletal symptoms

Note: *Exceptions to the reading-frame hypothesis have been reported, including BMD in patients with frame-shift mutations and DMD in patients with in-frame mutations. Such cases may represent up to 10% of all DMD gene mutations.*

Table S2. FDA-approved oligonucleotide therapy (ASOs) for DMD.

Drug (brand name)/ company	Year of the approval	Mechanism of action	Eligible patients	Description of the study (example of the clinical trial)	Outcome of the study	Dose used in DMD patients	References
**Eteplirsen (Exondys 51)**<br><br>Sarepta Therapeutics	2016	*DMD* exon-51 skipping	14%	NCT02420379<br><br>A 96-week-long study performed on 25 DMD patients amenable to exon 51 skipping	Only modest increases in dystrophin restoration	30 mg/kg of patient weight, administered once weekly by intravenous infusion for 96 weeks	Dowling (2016)[1]<br>Lim et al. (2017)[2]
**Golodirsen (Vyondys 53)**<br><br>Sarepta Therapeutics	2019	*DMD* exon-53 skipping	8%	NCT02310906<br><br>Phase 1/2 study: 1st part: a 12-week, randomized, double-blind, placebo-controlled, dose-titration study followed by a 9-week safety review involving 12 DMD patients, with 8 patients receiving Vyondys 53 and 4 receiving placebo.<br><br>2nd part: a 168-week, open-label evaluation of golodirsen 30 mg/kg in 12 patients enrolled in the 1st part, and 13 additional patients.	Dystrophin levels, detected by western blot, increased on average, from 0.095% of normal at baseline to 1.019% of normal after 48 weeks of treatment with the drug	30 mg/kg of patient's weight by intravenous infusion once per week	Servais et al. (2022)[3]<br>Sheikh and Yokota (2022)[4]
**Viltolarsen (Viltepso)**<br><br>NS Pharma	2020	*DMD* exon-53 skipping	8-10%	NCT02740972<br><br>An initial Phase 2, two-period, randomized, dose-finding trial consisted of a	The increase in dystrophin production was established in one of those two studies,	80 mg/kg of patient weight once weekly by intravenous infusion	Clemens et al. (2022)[5]<br>Dhillon (2020)[6]<br>Roshmi and Yokota (2019)[7]

				4-week double-blinded, placebo-controlled period for the safety of viltolarsen followed by a 20-week open-label period to evaluate the efficacy, safety, and tolerability of viltolarsen<br><br>NCT03167255<br><br>Extension Study to evaluate the clinical efficacy and safety of viltolarsen over a longer time (up to an additional 192 weeks)	which included 16 DMD patients, with 8 patients receiving Viltepso at the recommended dose. In this study, dystrophin levels increased, on average, from 0.6% of normal at baseline to 5.9% of normal at week 25		
**Casimersen (Amondys 45)**<br><br>Sarepta Therapeutics	2021	*DMD* exon-45 skipping	9%	NCT02500381<br><br>a double-blind, placebo-controlled study<br>43 male patients, between 7 and 20 years of age, were randomized 2:1 to receive either intravenous Amondys 45 (30 mg/kg) or placebo	Mean dystrophin levels (measured as a percentage of normal, i.e., in healthy subjects) increased from 0.93% at baseline to 1.74% at week 48 among patients treated with IV casimersen 30 mg/kg once weekly	30 mg/kg of patient weight once weekly by intravenous infusion	Assefa et al. (2024)[8]<br>Shirley (2021)[9]

Table S3. Selected completed or ongoing clinical trials for DMD utilizing adeno-associated virus (AAV)-mediated small dystrophin constructs. Three major pharmaceutical companies, Sarepta Therapeutics, Solid Biosciences, and Pfizer, are testing the potential of µDys in humans (based on clinicaltrials.gov). Additionally, Genethon, a gene therapy research organization created by AFM-Telethon, is conducting trials with µDys (registered at the EU Clinical Trials Register; www.clinicaltrialsregister.eu). In all trials, the vectors were administered as a single intravenous infusion.

SAREPTA THERAPEUTICS					
**OFFICIAL TRIAL TITLE (ACRONYM)**	Systemic Gene Delivery Phase I/IIa Clinical Trial for Duchenne Muscular Dystrophy Using rAAVrh74.MHCK7. Micro-dystrophin (microDys-IV-001) (Study 101)	A Multicenter, Randomized, Double-Blind, Placebo-Controlled Trial for Duchenne Muscular Dystrophy Using SRP-9001 (Study 102)	An Open-Label, Systemic Gene Delivery Study Using Commercial Process Material to Evaluate the Safety of and Expression From SRP-9001 in Subjects With Duchenne Muscular Dystrophy (ENDEAVOR; Study 103)	A Phase 3 Multinational, Randomized, Double-Blind, Placebo-Controlled Systemic Gene Delivery Study to Evaluate the Safety and Efficacy of SRP-9001 in Subjects With Duchenne Muscular Dystrophy (EMBARK; Study SRP 301)	An Open-Label, Systemic Gene Delivery Study to Evaluate the Safety, Tolerability and Expression of Delandistrogene Moxeparvovec Following Plasmapheresis in Subjects With Duchenne Muscular Dystrophy and Pre-existing Antibodies to AAVrh74
**TRIAL NUMBER**	NCT03375164	NCT03769116	NCT04626674	NCT05096221	NCT06597656
**STATUS**	completed	completed	active, not recruiting	completed	recruiting
**TREATMENT NAME**	SRP-9001	SRP-9001	SRP-9001	SRP-9001	SRP-9001
**AAV SEROTYPE**	rAAVrh74	rAAVrh74	rAAVrh74	rAAVrh74	rAAVrh74
**ENROLLMENT (actual)**	4 participants	41 participants	55 participants	126 participants	16 participants (estimated)
**AGES ELIGIBLE FOR STUDY**	3 months to 7 years	4 years to 7 years	2 years and older	4 years to 7 years	4 years to 8 years

SOLID BIOSCIENCES		
**OFFICIAL TRIAL TITLE (ACRONYM)**	A Randomized, Controlled, Open-label, Single-ascending Dose, Phase I/II Study to Investigate the Safety and Tolerability, and Efficacy of Intravenous SGT-001 in Male Adolescents and Children With Duchenne Muscular Dystrophy	A Phase 1/2, Multicenter, Open-Label Study to Investigate the Safety, Tolerability, and Efficacy of a Single Intravenous Dose of SGT-003 in Ambulant Males With Duchenne Muscular Dystrophy (INSPIRE DUCHENNE)
**TRIAL NUMBER**	NCT03368742	NCT06138639
**STATUS**	active, not recruiting	recruiting
**TREATMENT NAME**	SGT-001	SGT-003
**AAV SEROTYPE**	AAV9	AAV-SLB101
**ENROLLMENT (actual)**	12 participants	43 participants (estimated)
**AGES ELIGIBLE FOR STUDY**	4 years to 17 years	4 years to 11 years

PFIZER			
**OFFICIAL TRIAL TITLE (ACRONYM)**	A Phase 1b Multicenter, Open-Label, Single Ascending Dose Study To Evaluate The Safety And Tolerability of PF-06939926 in Ambulatory and Non-Ambulatory Subjects with Duchenne Muscular Dystrophy	A Phase 2, Multicenter, Single-Arm Study to Evaluate the Safety and Dystrophin Expression after Fordadistrogene Movaparvovec (PF-06939926) Administration in Male Participants with Early Stage Duchenne Muscular Dystrophy	A Phase 3, Multicenter, Randomized, Double-Blind, Placebo Controlled Study to Evaluate the Safety and Efficacy of PF-06939926 for the Treatment of Duchenne Muscular Dystrophy (CIFFREO)
**TRIAL NUMBER**	NCT03362502	NCT05429372	NCT04281485
**STATUS**	active, not recruiting	active, not recruiting	active, not recruiting
**TREATMENT NAME**	PF-06939926	PF-06939926	PF-06939926
**AAV SEROTYPE**	AAV9	AAV9	AAV9
**ENROLLMENT (actual)**	22 participants	10 participants	122 participants
**AGES ELIGIBLE FOR STUDY**	4 years and older	2 years to 3 years	4 years to 7 years

REGENXBIO	
**OFFICIAL TRIAL TITLE (ACRONYM)**	A Phase 1/2 Open-label, Dose Escalation and Dose Expansion Study to Evaluate the Safety, Tolerability, Pharmacodynamics, and Pharmacokinetics of Intravenous RGX-202 Gene Therapy in Males With Duchenne Muscular Dystrophy (DMD) (AFFINITY DUCHENNE)
**TRIAL NUMBER**	NCT05693142
**STATUS**	recruiting
**TREATMENT NAME**	RGX-202
**AAV SEROTYPE**	AAV8
**ENROLLMENT (actual)**	15 participants (estimated)
**AGES ELIGIBLE FOR STUDY**	1 Year to 11 Years

GENETHON	
**OFFICIAL TRIAL TITLE (ACRONYM)**	A phase I/II/III study with a dose determination part followed by an efficacy and safety evaluation of selected dose part and then by a long-term follow-up part in ambulant boys aged 6 to 10 years with DMD (GNT-016-MYDF)
**TRIAL NUMBER**	EudraCT Number: 2020-002093-27
**STATUS**	recruiting
**TREATMENT NAME**	GNT0004
**AAV SEROTYPE**	AAV8
**ENROLLMENT (actual)**	~50 participants
**AGES ELIGIBLE FOR STUDY**	6 years to 10 years

Abbreviations: *NCT number – ClinicalTrials.gov identifier; GNT0004 – AAV8-µDys; rAAVrh74.MHCK7 – delandistrogene moxeparvovec, SRP-9001; PF-06939926 – fordadistrogene movaparvovec; SRP-9001 – delandistrogene moxeparvovec*

Table S4. Examples of CRISPR/Cas-based approaches to treat DMD in animal models.

CRISPR/Cas system	Animal model/ *DMD* mutation	Genetic effect	Delivery tool/ mode of injection	Global effects	References
**CRISPR/Cas9 editing by NHEJ or HDR in zygote**					
SpCas9	*mdx* mice	Correction of point mutation in exon 23	Injection into zygote; mRNA of SpCas9, sgRNA and single-stranded DNA repair template	Mosaic animals with restoration of dystrophin expression from 2 to 100%; decreased muscle damage	Long et al. (2014)[10]
LbCpf1 *(Lachnospiraceae bacterium)*	*mdx* mice	Correction of point mutation in exon 23	Injection into zygote: mRNA of LbCpf1, sgRNA and repair template	Restoration of dystrophin expression; improved muscle strength; correction also in germ cells	Zhang et al. (2017)[11]
**CRISPR/Cas9 editing - exon skipping or exon reframing**					
SaCas9	*mdx* mice	Deletion of exon 23	AAV9 – two vectors (SPCas9 and sgRNA); i.m. and i.v.,	Restoration of dystrophin, improved muscle function	Nelson et al. (2016)[12]
SaCas9	*mdx* mice	Deletion of exon 23	AAV9 – single or dual system; i.m.; i.p. (P3)	Restoration of dystrophin (deletion of exon 23 in 5% (heart) to 20% (muscles), improved muscle function; modification of satellite cells; dual AAV9 system more effective	Tabebordbar et al. (2016)[13]
SpCas9	*mdx* mice	Deletion of exon 23	AAV9 - two vectors (SPCas9 and sgRNA, i.m (postnatal day 12 – P12), i.v. (P18), i.p. (P1)	Restoration of dystrophin in skeletal muscles and heart; increased muscle force	Long et al. (2016)[14]
SaCas9	*mdx/Utr*$^{+/-}$ neonates (P1-P3) or adult	Exons 20 to 23 excision	Adenovirus and AAVrh74	Restoration of dystrophin expression to 40%; improvement of cardiac function	El Refaey et al. (2017)[15]

SpCas9 - dual vector system for HDR repair<br><br>SaCas9 - single vector	*mdx*^{4cv} mice, nonsense mutation in exon 53	Deletion of exons 52 and 53 or gene correction by HDR	AAV with muscle-specific promoter; dual vector system with SPCas9, shgRNA, and HDR cassette;<br>Single vector with SaCas9 and sgRNA; i.m. and i.v.	Up to 70% of muscle dystrophin expression restored; dystrophin restored in the heart	Bengtsson et al. (2017)[16]
SpCas9	ΔEx50 mice generated by CRISPR/Cas9 deletion	Exon 51 skipping	AAV9,<br>i.m. 5×10^{10} vg;<br>i.p. 2.67×10^{13} vg/kg	Up to 90% dystrophin restoration in muscles and heart; enhanced grip strength	Amoasii et al. (2017)[17]
SpCas9	ΔEx44 mice generated by CRISPR/Cas9	Exon 43 or 45 skipping or reframing	AAV9, i.m. and i.p,	Restoration of dystrophin expression; systemic delivery restored in more than 90% myofibres and cardiomyocytes; better edition with a higher quantity of sgRNAs present	Min et al. (2019)[18]
SpCas9	ΔEx43 & ΔEx45 mice generated by CRISPR/Cas9<br><br>ΔEx52 mice	Exon 44 skipping or reframing<br><br>Exon 53 skipping or reframing	AAV9; dual system (SPCas9 in ssAAV vector; sgRNA in scAAV vector); CKE8 muscle regulatory cassette; i.m.	The same approach restored dystrophin expression in two DMD mouse models (by exon skipping in ΔEx43 and by skipping and reframing of exon 44 in ΔEx45; about 20% dystrophin restoration; restoration of dystrophin in ΔEx45 more efficient (skipping and reframing)<br><br>About 50% dystrophin restoration	Min et al. (2020)[19]
SaCas9	*mdx*^{4cv} mice	Deletion of exons 52 and 53	AAV6	Dystrophin expression persisted for 18 weeks in cardiac but not skeletal muscles	Bengtsson et al. (2021)[20]

SpCas9	*mdx* mice	Deletion of exons 23 and 24	Extracellular vesicles, SpCas9 protein and gRNA	Restored dystrophin expression in about 20% positive fibers	Majeau et al. (2022)[21]
SpCas9	Three canine models: GRMD, WCMD, and LRMD	Targeting introns 5 and 8<br><br>Blocking splicing of the new exon by targeting SA or ESE	AAV8, i.m. or i.v.	Wide dystrophin rescue, but CTL immune response against Cas9	Hakim et al. (2021)[22]
SaCas9	Dogs, 3 and 8-year-old	Exon 6-8 excision (105 kb region)	AAV6	Up to 4% dystrophin transcript expressed; DMD gene editing compared with microdystrophin expression – the latter provided a greater effect	Bengtsson et al. (2022)[23]
SpCas9	Beagle dog ΔEx50	Exon 51 skipping or reframing (by A insertion)	AAV9, i.m. and i.v.; 2×10^{13} and 10^{14} vg/kg	Restored dystrophin expression (3-92%); better effect with a higher dose	Amoasii et al. (2018)[24]
SpCas9	Pig *DMD* ΔEx52; piglets 10-14d	Exon 51 excision	AAV9, i.m (2×10^{13}) and i.v. (2×10^{14} vg/kg) intein-split Cas9	Widespread dystrophin in skeletal muscles, diaphragm & heart; prolonged survival and reduced arrhythmogenic vulnerability	Moretti et al. (2020)[25]
**Adenine base editing**					
SpCas9-based nickase	*mdx* mice with exon 23 (mice generated previously by CBE [26])	Point mutation in exon 20	Deoxyadenosine deaminase Dual AAV trans-splicing vectors – i.m.	Correction of mutation - dystrophin expression in 17% fibers; sarcolemmal localisation of nNOS restored; no off-target effects	Ryu et al., (2018)[27]
SpCas9-based nickase	ΔEx51 mice	Splice donor site	AAV9; split-intein approach (10^{11} vg/muscle – corresponds to 10^{16} vg/kg	Disease correction by exon 50 skipping	Chemello et al. (2021)[28]
SpCas9-based nickase	*mdx*[4cv] mice	Exon 53 point mutation	AAV9 systemic delivery; split-intein approach	Dystrophin restoration, at 10 months near complete rescue	Xu et al. (2021)[29]

				in the heart, up to 15% in the skeletal muscles	
SpCas9-based nickase	ΔEx44 mice	Splicing site in exon 45	AAV9, trans-splicing intein-based two AAV vectors; CKE8 muscle-specific promoters; i.v. 1.5×10^{14} vg/kg	Exon 45 skipping; restoration of dystrophin expression, improved grip strength	Chai et al. (2023)[30]
SpCas9-based nickase	Humanized DMD mice model (exons 50 and 51 replaced with human Ex 50)	Splice site of exon 50	AAV (serotype not indicated); trans-splicing intein-based; two AAV vectors i.p.	Dystrophin restoration; editing noted in satellite cells; exon-skipping in heart 2x higher than in skeletal muscles (75% dystrophin vs 50% restored); effect lasted for 10 months; decreased heart fibrosis; improved skeletal muscle (grip test)	Lin et al. (2024)[31]
SpCas9-based nickase	Humanized DMD mice model $DMD^{Ex30mut}$<br><br>Humanized DMD mice model $DMD^{Ex23mut}$	The nonsense point mutation (c.4174C> T) in exon 30; correction A to G<br><br>The nonsense point mutation (c.2977C>T) in exon 23	AAV9, i.m. 5×10^{11} vg/leg<br><br>i.p., 1×10^{11} vg delivery to neonatal mice (at postnatal day 3)	Efficient restoration of dystrophin expression in heart and skeletal muscle (more efficient in the heart); decreased heart and muscle fibrosis; improvement in muscle function<br><br>Correction of a point mutation in Ex30 more efficient than in Ex23	Jin et al. (2024)[32]
**Cytidine base editing**					
SaCas9-based nickase	DMDE4* mice created by introducing a 4-bp deletion in exon 4	5'-splice site of exon 4 (GT > AT)	AAV9, two vectors: SaCas9 fused with cytidine deaminase and uracil glycosylase inhibitor, and the second with three copies of sgRNAs; 10^{12} vg per mouse, i.p., neonatal mice	>50% skipping exon 4; restoration of dystrophin in the heart (up to 90%) and skeletal muscle; increased life span; effect lasted more than 1 year	Li et al. (2021)[33]

IscB-M16*-CBE	ΔEx50 mice, 3-week-old	CBE disrupted AG splicing acceptor adjacent to exon 50 to cause exon 51 skipping	AAV9 – single vector with IscB-M16*-CBE (4.0 kb); i.m. 2.5×10^{11} vg/kg	Up to 30% level of exon 51 skipping; restoration of dystrophin level to 40% of WT controls	Xiao et al. (2024)[34]
SpCas9-based nickase	ΔEx54 humanized mice, 3-week-old	Splice acceptor site of exon 55, results in the splicing of exons 53 to 56	AAV9, split-intein, TadA CBE from *Acinetobacter junii*; i.m.	Skipping exon 55; 60% dystrophin restored in tibialis muscle 6 weeks after i.m. injection	Li et al. (2024)[35]
**RNA base editing**					
EcCas6E cERBE (RNA-based editor)	DMD Q1392X humanized mice, 8-week-old	c.4174C>T mutat8-week-oldn 30 (Q1382X)	AAV9, single vector with EcCasE6 and adenine base editor; i.m.	~70% of fibers with recovered dystrophin expression 3 weeks after base editing	Wang et al. (2023)[36]

Abbreviations: *A – adenosine; ABE – adenosine base editor, CBE – cytosine base editor, CTL – cytotoxic T-lymphocyte; ESE – exonic splicing enhancer; GRMD – golden retriever muscular dystrophy; LRMD – labrador retriever muscular dystrophy dog; i.m. – intramuscular, i.p. – intraperitoneal; i.v. – intravenous injections; SA – splice acceptor; SaCas9 – Staphylococcus aureus Cas9; SpCas9 – Streptococcus pyogenes Cas9; split-intein – two segments of editing cassette are delivered with two AAV vectors; WCMD – Welsh corgi muscular dystrophy dog; vg/kg – vector genomes per kilogram of body weight*

References

1. Dowling, J.J. (2016). Eteplirsen therapy for Duchenne muscular dystrophy: skipping to the front of the line. Nat Rev Neurol *12*, 675–676. https://doi.org/10.1038/nrneurol.2016.180.

2. Lim, K.R.Q., Maruyama, R., and Yokota, T. (2017). Eteplirsen in the treatment of Duchenne muscular dystrophy. Drug Des Devel Ther *11*, 533–545. https://doi.org/10.2147/DDDT.S97635.

3. Servais, L., Mercuri, E., Straub, V., Guglieri, M., Seferian, A.M., Scoto, M., Leone, D., Koenig, E., Khan, N., Dugar, A., et al. (2022). Long-Term Safety and Efficacy Data of Golodirsen in Ambulatory Patients with Duchenne Muscular Dystrophy Amenable to Exon 53 Skipping: A First-in-human, Multicenter, Two-Part, Open-Label, Phase 1/2 Trial. Nucleic Acid Ther *32*, 29–39. https://doi.org/10.1089/nat.2021.0043.

4. Sheikh, O., and Yokota, T. (2022). Pharmacology and toxicology of eteplirsen and SRP-5051 for DMD exon 51 skipping: an update. Arch Toxicol *96*, 1–9. https://doi.org/10.1007/s00204-021-03184-z.

5. Clemens, P.R., Rao, V.K., Connolly, A.M., Harper, A.D., Mah, J.K., McDonald, C.M., Smith, E.C., Zaidman, C.M., Nakagawa, T., CINRG DNHS Investigators, et al. (2022). Long-Term Functional Efficacy and Safety of Viltolarsen in Patients with Duchenne Muscular Dystrophy. J Neuromuscul Dis *9*, 493–501. https://doi.org/10.3233/JND-220811.

6. Dhillon, S. (2020). Viltolarsen: First Approval. Drugs. https://doi.org/10.1007/s40265-020-01339-3.

7. Roshmi, R.R., and Yokota, T. (2019). Viltolarsen for the treatment of Duchenne muscular dystrophy. Drugs Today (Barc) *55*, 627–639. https://doi.org/10.1358/dot.2019.55.10.3045038.

8. Assefa, M., Gepfert, A., Zaheer, M., Hum, J.M., and Skinner, B.W. (2024). Casimersen (AMONDYS 45™): An Antisense Oligonucleotide for Duchenne Muscular Dystrophy. Biomedicines *12*, 912. https://doi.org/10.3390/biomedicines12040912.

9. Shirley, M. (2021). Casimersen: First Approval. Drugs *81*, 875–879. https://doi.org/10.1007/s40265-021-01512-2.

10. Long, C., McAnally, J.R., Shelton, J.M., Mireault, A.A., Bassel-Duby, R., and Olson, E.N. (2014). Prevention of muscular dystrophy in mice by CRISPR/Cas9-mediated editing of germline DNA. Science *345*, 1184–1188. https://doi.org/10.1126/science.1254445.

11. Zhang, Y., Long, C., Li, H., McAnally, J.R., Baskin, K.K., Shelton, J.M., Bassel-Duby, R., and Olson, E.N. (2017). CRISPR-Cpf1 correction of muscular dystrophy mutations in human cardiomyocytes and mice. Science Advances *3*, e1602814. https://doi.org/10.1126/sciadv.1602814.

12. Nelson, C.E., Hakim, C.H., Ousterout, D.G., Thakore, P.I., Moreb, E.A., Castellanos Rivera, R.M., Madhavan, S., Pan, X., Ran, F.A., Yan, W.X., et al. (2016). In vivo genome editing improves muscle function in a mouse model of Duchenne muscular dystrophy. Science *351*, 403–407. https://doi.org/10.1126/science.aad5143.

13. Tabebordbar, M., Zhu, K., Cheng, J.K.W., Chew, W.L., Widrick, J.J., Yan, W.X., Maesner, C., Wu, E.Y., Xiao, R., Ran, F.A., et al. (2016). In vivo gene editing in dystrophic mouse muscle and muscle stem cells. Science *351*, 407–411. https://doi.org/10.1126/science.aad5177.

14. Long, C., Amoasii, L., Mireault, A.A., McAnally, J.R., Li, H., Sanchez-Ortiz, E., Bhattacharyya, S., Shelton, J.M., Bassel-Duby, R., and Olson, E.N. (2016). Postnatal genome editing partially restores dystrophin expression in a mouse model of muscular dystrophy. Science *351*, 400–403. https://doi.org/10.1126/science.aad5725.

15. El Refaey, M., Xu, L., Gao, Y., Canan, B.D., Adesanya, T.M.A., Warner, S.C., Akagi, K., Symer, D.E., Mohler, P.J., Ma, J., et al. (2017). In Vivo Genome Editing Restores Dystrophin Expression and Cardiac Function in Dystrophic Mice. Circ Res *121*, 923–929. https://doi.org/10.1161/CIRCRESAHA.117.310996.

16. Bengtsson, N.E., Hall, J.K., Odom, G.L., Phelps, M.P., Andrus, C.R., Hawkins, R.D., Hauschka, S.D., Chamberlain, J.R., and Chamberlain, J.S. (2017). Muscle-specific CRISPR/Cas9 dystrophin gene editing ameliorates pathophysiology in a mouse model for Duchenne muscular dystrophy. Nat Commun *8*, 14454. https://doi.org/10.1038/ncomms14454.

17. Amoasii, L., Long, C., Li, H., Mireault, A.A., Shelton, J.M., Sanchez-Ortiz, E., McAnally, J.R., Bhattacharyya, S., Schmidt, F., Grimm, D., et al. (2017). Single-cut genome editing restores dystrophin expression in a new mouse model of muscular dystrophy. Sci Transl Med *9*, eaan8081. https://doi.org/10.1126/scitranslmed.aan8081.

18. Min, Y.-L., Li, H., Rodriguez-Caycedo, C., Mireault, A.A., Huang, J., Shelton, J.M., McAnally, J.R., Amoasii, L., Mammen, P.P.A., Bassel-Duby, R., et al. (2019). CRISPR-Cas9 corrects Duchenne muscular dystrophy exon 44 deletion mutations in mice and human cells. Sci Adv *5*, eaav4324. https://doi.org/10.1126/sciadv.aav4324.

19. Min, Y.-L., Chemello, F., Li, H., Rodriguez-Caycedo, C., Sanchez-Ortiz, E., Mireault, A.A., McAnally, J.R., Shelton, J.M., Zhang, Y., Bassel-Duby, R., et al. (2020). Correction of Three Prominent Mutations in Mouse and Human Models of Duchenne Muscular Dystrophy by Single-Cut Genome Editing. Mol Ther *28*, 2044–2055. https://doi.org/10.1016/j.ymthe.2020.05.024.

20. Bengtsson, N.E., Tasfaout, H., Hauschka, S.D., and Chamberlain, J.S. (2021). Dystrophin Gene-Editing Stability Is Dependent on Dystrophin Levels in Skeletal but Not Cardiac Muscles. Mol Ther *29*, 1070–1085. https://doi.org/10.1016/j.ymthe.2020.11.003.

21. Majeau, N., Fortin-Archambault, A., Gérard, C., Rousseau, J., Yaméogo, P., and Tremblay, J.P. (2022). Serum extracellular vesicles for delivery of CRISPR-CAS9 ribonucleoproteins to modify the dystrophin gene. Mol Ther *30*, 2429–2442. https://doi.org/10.1016/j.ymthe.2022.05.023.

22. Hakim, C.H., Kumar, S.R.P., Pérez-López, D.O., Wasala, N.B., Zhang, D., Yue, Y., Teixeira, J., Pan, X., Zhang, K., Million, E.D., et al. (2021). Cas9-specific immune responses compromise local and systemic AAV CRISPR therapy in multiple dystrophic canine models. Nat Commun *12*, 6769. https://doi.org/10.1038/s41467-021-26830-7.

23. Bengtsson, N.E., Crudele, J.M., Klaiman, J.M., Halbert, C.L., Hauschka, S.D., and Chamberlain, J.S. (2022). Comparison of dystrophin expression following gene editing and gene replacement in an aged preclinical DMD animal model. Mol Ther *30*, 2176–2185. https://doi.org/10.1016/j.ymthe.2022.02.003.

24. Amoasii, L., Hildyard, J.C.W., Li, H., Sanchez-Ortiz, E., Mireault, A., Caballero, D., Harron, R., Stathopoulou, T.-R., Massey, C., Shelton, J.M., et al. (2018). Gene editing restores dystrophin expression in a canine model of Duchenne muscular dystrophy. Science *362*, 86–91. https://doi.org/10.1126/science.aau1549.

25. Moretti, A., Fonteyne, L., Giesert, F., Hoppmann, P., Meier, A.B., Bozoglu, T., Baehr, A., Schneider, C.M., Sinnecker, D., Klett, K., et al. (2020). Somatic gene editing ameliorates skeletal and cardiac muscle failure in pig and human models of Duchenne muscular dystrophy. Nat. Med. *26*, 207–214. https://doi.org/10.1038/s41591-019-0738-2.

26. Kim, K., Ryu, S.-M., Kim, S.-T., Baek, G., Kim, D., Lim, K., Chung, E., Kim, S., and Kim, J.-S. (2017). Highly efficient RNA-guided base editing in mouse embryos. Nat Biotechnol *35*, 435–437. https://doi.org/10.1038/nbt.3816.

27. Ryu, S.-M., Koo, T., Kim, K., Lim, K., Baek, G., Kim, S.-T., Kim, H.S., Kim, D.-E., Lee, H., Chung, E., et al. (2018). Adenine base editing in mouse embryos and an adult mouse model of Duchenne muscular dystrophy. Nat Biotechnol *36*, 536–539. https://doi.org/10.1038/nbt.4148.

28. Chemello, F., Chai, A.C., Li, H., Rodriguez-Caycedo, C., Sanchez-Ortiz, E., Atmanli, A., Mireault, A.A., Liu, N., Bassel-Duby, R., and Olson, E.N. (2021). Precise correction of Duchenne muscular dystrophy exon deletion mutations by base and prime editing. Sci Adv *7*, eabg4910. https://doi.org/10.1126/sciadv.abg4910.

29. Xu, L., Zhang, C., Li, H., Wang, P., Gao, Y., Mokadam, N.A., Ma, J., Arnold, W.D., and Han, R. (2021). Efficient precise in vivo base editing in adult dystrophic mice. Nat Commun *12*, 3719. https://doi.org/10.1038/s41467-021-23996-y.

30. Chai, A.C., Chemello, F., Li, H., Nishiyama, T., Chen, K., Zhang, Y., Sánchez-Ortiz, E., Alomar, A., Xu, L., Liu, N., et al. (2023). Single-swap editing for the correction of common Duchenne muscular dystrophy mutations. Mol Ther Nucleic Acids *32*, 522–535. https://doi.org/10.1016/j.omtn.2023.04.009.

31. Lin, J., Jin, M., Yang, D., Li, Z., Zhang, Y., Xiao, Q., Wang, Y., Yu, Y., Zhang, X., Shao, Z., et al. (2024). Adenine base editing-mediated exon skipping restores dystrophin in humanized Duchenne mouse model. Nat Commun *15*, 5927. https://doi.org/10.1038/s41467-024-50340-x.

32. Jin, M., Lin, J., Li, H., Li, Z., Yang, D., Wang, Y., Yu, Y., Shao, Z., Chen, L., Wang, Z., et al. (2024). Correction of human nonsense mutation via adenine base editing for Duchenne muscular dystrophy treatment in mouse. Mol Ther Nucleic Acids *35*, 102165. https://doi.org/10.1016/j.omtn.2024.102165.

33. Li, J., Wang, K., Zhang, Y., Qi, T., Yuan, J., Zhang, L., Qiu, H., Wang, J., Yang, H.-T., Dai, Y., et al. (2021). Therapeutic Exon Skipping Through a CRISPR-Guided Cytidine Deaminase Rescues Dystrophic Cardiomyopathy in Vivo. Circulation *144*, 1760–1776. https://doi.org/10.1161/CIRCULATIONAHA.121.054628.

34. Xiao, Q., Li, G., Han, D., Wang, H., Yao, M., Ma, T., Zhou, J., Zhang, Y., Zhang, X., He, B., et al. (2024). Engineered IscB-ωRNA system with expanded target range for base editing. Nat Chem Biol. https://doi.org/10.1038/s41589-024-01706-1.

35. Li, G., Dong, X., Luo, J., Yuan, T., Li, T., Zhao, G., Zhang, H., Zhou, J., Zeng, Z., Cui, S., et al. (2024). Engineering TadA ortholog-derived cytosine base editor without motif preference and adenosine activity limitation. Nat Commun *15*, 8090. https://doi.org/10.1038/s41467-024-52485-1.

36. Wang, X., Zhang, R., Yang, D., Li, G., Fan, Z., Du, H., Wang, Z., Liu, Y., Lin, J., Wu, X., et al. (2023). Develop a Compact RNA Base Editor by Fusing ADAR with Engineered EcCas6e. Adv Sci (Weinh) *10*, e2206813. https://doi.org/10.1002/advs.202206813.
